# Next-Generation Sequencing Profiles of the Methylome and Transcriptome in Peripheral Blood Mononuclear Cells of Rheumatoid Arthritis

**DOI:** 10.3390/jcm8091284

**Published:** 2019-08-22

**Authors:** Chia-Chun Tseng, Yuan-Zhao Lin, Chia-Hui Lin, Ruei-Nian Li, Chang-Yi Yen, Hua-Chen Chan, Wen-Chan Tsai, Tsan-Teng Ou, Cheng-Chin Wu, Wan-Yu Sung, Jeng-Hsien Yen

**Affiliations:** 1 Graduate Institute of Clinical Medicine, College of Medicine, Kaohsiung Medical University, Kaohsiung 80708, Taiwan; 2 Department of Internal Medicine, Kaohsiung Municipal Ta-Tung Hospital, Kaohsiung 80145, Taiwan; 3 Department of Biomedical Science and Environmental Biology, College of Life Science, Kaohsiung Medical University, Kaohsiung 80708, Taiwan; 4 Department of Internal Medicine, National Cheng Kung University Hospital, Tainan 70403, Taiwan; 5 Division of Rheumatology, Department of Internal Medicine, Kaohsiung Medical University Hospital, Kaohsiung 80754, Taiwan; 6 Institute of Medical Science and Technology, National Sun Yat-Sen University, Kaohsiung 80424, Taiwan; 7 Department of Biological Science and Technology, National Chiao-Tung University, Hsinchu 30010, Taiwan

**Keywords:** rheumatoid arthritis, methylation, next-generation sequencing

## Abstract

Using next-generation sequencing to decipher methylome and transcriptome and underlying molecular mechanisms contributing to rheumatoid arthritis (RA) for improving future therapies, we performed methyl-seq and RNA-seq on peripheral blood mononuclear cells (PBMCs) from RA subjects and normal donors. Principal component analysis and hierarchical clustering revealed distinct methylation signatures in RA with methylation aberrations noted across chromosomes. Methylation alterations varied with CpG features and genic characteristics. Typically, CpG islands and CpG shores were hypermethylated and displayed the greatest methylation variance. Promoters were hypermethylated and enhancers/gene bodies were hypomethylated, with methylation variance associated with expression variance. RA genetically associated genes preferentially displayed differential methylation and differential expression or interacted with differentially methylated and differentially expressed genes. These differentially methylated and differentially expressed genes were enriched with several signaling pathways and disease categories. 10 genes (*CD86, RAB20, XAF1*, *FOLR3, LTBR*, *KCNH8*, *DOK7, PDGFA, PITPNM2, CELSR1*) with concomitantly differential methylation in enhancers/promoters/gene bodies and differential expression in B cells were validated. This integrated analysis of methylome and transcriptome identified novel epigenetic signatures associated with RA and highlighted the interaction between genetics and epigenetics in RA. These findings help our understanding of the pathogenesis of RA and advance epigenetic studies in regards to the disease.

## 1. Introduction

Rheumatoid arthritis (RA) is an autoimmune disease manifested by sustained chronic inflammation resulting in joint damage and severe disability. Numerous therapies based on our knowledge of RA were developed over the past two decades and helped improve outcomes for those suffering from the disease [1]. Despite the increasing number of treatment strategies, many patients are refractory to their current treatments, some patients see their clinical response diminish, while others suffer from adverse events from therapy. As such, there is a necessity to develop an improved strategy to treat RA [2].

Engrafting peripheral blood mononuclear cells (PBMCs) of RA patients into severe combined immunodeficient (SCID) mice resulted in a reconstituted synovitis characteristic of human RA [3]. Additionally, these cells secreted numerous inflammatory cytokines, such as interleukin (IL)-6 and tumor necrosis factor-alpha (TNF-α) [4,5] which orchestrated inflammation, radiographic progression of RA and were, therefore, therapeutic targets of current RA management [6]. These findings highlight the critical role of PBMCs in RA pathogenesis. Therefore, a more comprehensive understanding of PBMCs in RA holds promise in unraveling the complexity of RA and identifying novel therapeutic targets.

In past decades, tremendous efforts have been devoted to exploring RA genetics. However, in recent years, DNA methylation is emerging as one key pathogenic player of RA. DNA methylation acts as a composite measure of environmental exposures [7], making it an intriguing candidate for the investigation of diseases that involve environmental factors, such as RA. Traditionally, DNA methylation has been thought of as being involved in gene silencing but recent work has shown a more complex picture [8]. Most studies investigating the role of DNA methylation in RA utilized the Illumina 450K microarray for methylation profiling and focused on methylation alone. Studies integrating DNA methylation with gene expression at a whole-genome manner to investigate the relationship between methylation, expression and RA, the associations of genomic contexts and DNA methylation and the interaction between differentially methylated genes and genetic at-risk loci in RA remain somewhat limited.

To decipher the methylation signatures involved in RA PBMCs, we performed next generation sequencing to compare the methylome and transcriptome landscape in PBMCs from RA patients and healthy donors, detect changes to the methylome and transcriptome, elucidate the relationship between methylation and expression and interaction between genetically associated genes and epigenetically associated genes. These results offered a map to the PBMCs methylome and shed light on the pathophysiology of RA.

## 2. Methods

**Figure 1:**
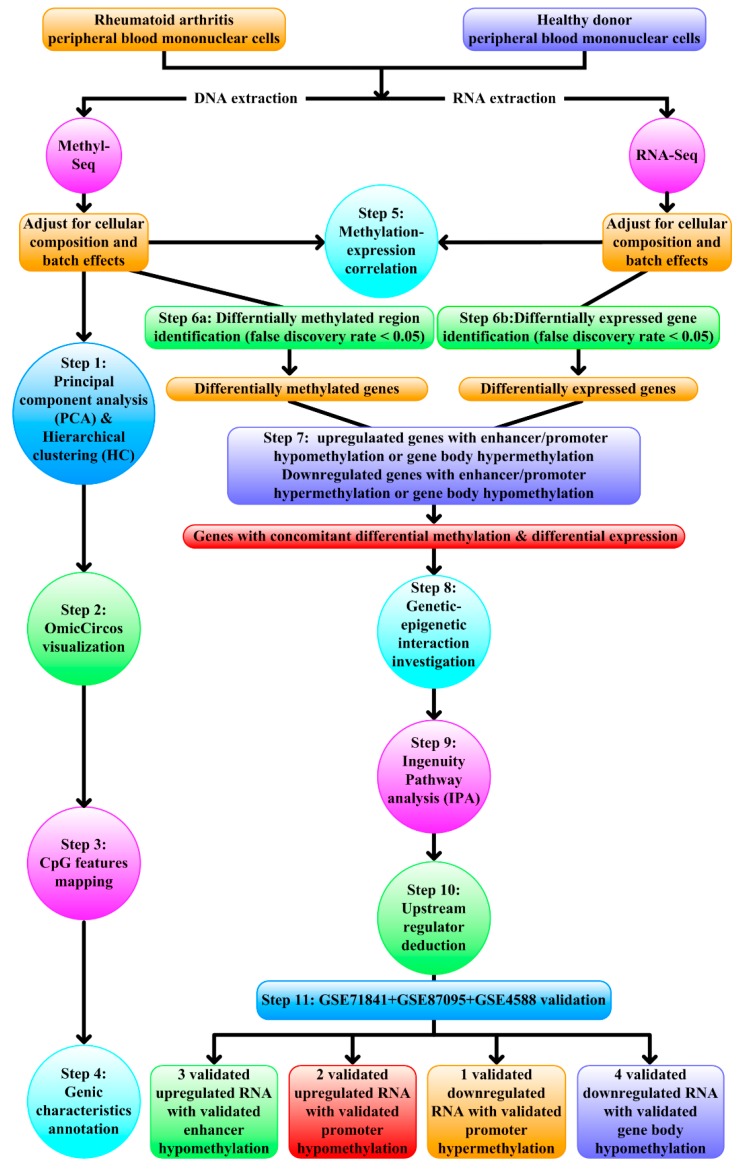
Schematic representation of the next-generation sequencing data analytical workflow. After adjusting for cellular composition and batch effects, methyl-seq data first underwent Principal component analysis (PCA) and hierarchical clustering (HC) (Step 1), OmicCircos visualization (Step 2), CpG features mapping (Step 3), and genic characteristics annotation (Step 4). Methylation and expression profiles were then integrated for methylation-expression correlation (Step 5). Differentially methylated genes (FDR < 0.05) and differentially expressed genes (FDR < 0.05) were identified (Step 6a–6b) and intersected to yield genes with concomitant expression and methylation changes in enhancer/promoter/gene body (Step 7). These differentially methylated and differentially expressed genes underwent genetic–epigenetic interaction investigation (Step 8), IPA (Step 9), and upstream regulator deduction (Step 10). GEO dataset validation (Step 11) confirmed concomitant differential methylation and expression of 10 genes.

After adjusting for cell types and batch effects (Figure 1), methylome data went through principal component analysis (PCA) and hierarchical clustering (HC) (Figure 1, Step 1), OmicCircos visualization (Figure 1, Step 2), CpG features mapping (Figure 1, Step 3), genic characteristics annotation (Figure 1, Step 4), integration with transcriptome for methylation-expression correlation (Figure 1, Step 5) and identification of concomitantly differentially methylated (false discovery rate (FDR) <0.05) and differentially expressed genes (FDR < 0.05) (Figure 1, Step 6–7). Genes with concomitantly differential methylation and differential expression underwent genetic–epigenetic interaction investigation (Figure 1, Step 8), Ingenuity Pathway Analysis (IPA) (Figure 1, Step 9), and upstream regulator deduction (Figure 1, Step 10). GEO dataset were downloaded for further validation (Figure 1, Step 11). For detailed methods, see Supplementary Files. The study was conducted in accordance with the Helsinki Declaration and was approved by the ethics committee of the Kaohsiung Medical University Hospital (KMUHIRB-G(II)-20180031). All subjects gave their informed consent for inclusion before they participated in the study.

## 3. Results

### 3.1. Differential Methylation of PBMCs in RA

After adjusting for cellular composition and batch effects, we first profiled DNA methylation alterations between RA and healthy donors with PCA and HC (Figure 1, Step 1). As shown in Figure S2a, RA samples were characterized by distinct methylation profiles compared with healthy donors. We also performed molecular stratification of samples using HC of methylation profiles (Figure S2b). Based on methylation profiles, two distinct groups were identified, with the results reaffirming the classification of RA and healthy donors.

### 3.2. Distribution of Methylation According to Genome Locations

For a visual representation of the analysis results, the R package Omiccircos was used to draw the circos-plot. Supplementary Figure S3 depicted the methylation differences between RA and healthy donors according to chromosome locations. Generally, methylation alterations were scattered across nuclear genomes. No clear concentration of methylation changes was identified. 

### 3.3. CpG Features Mapping

**Figure 2:**
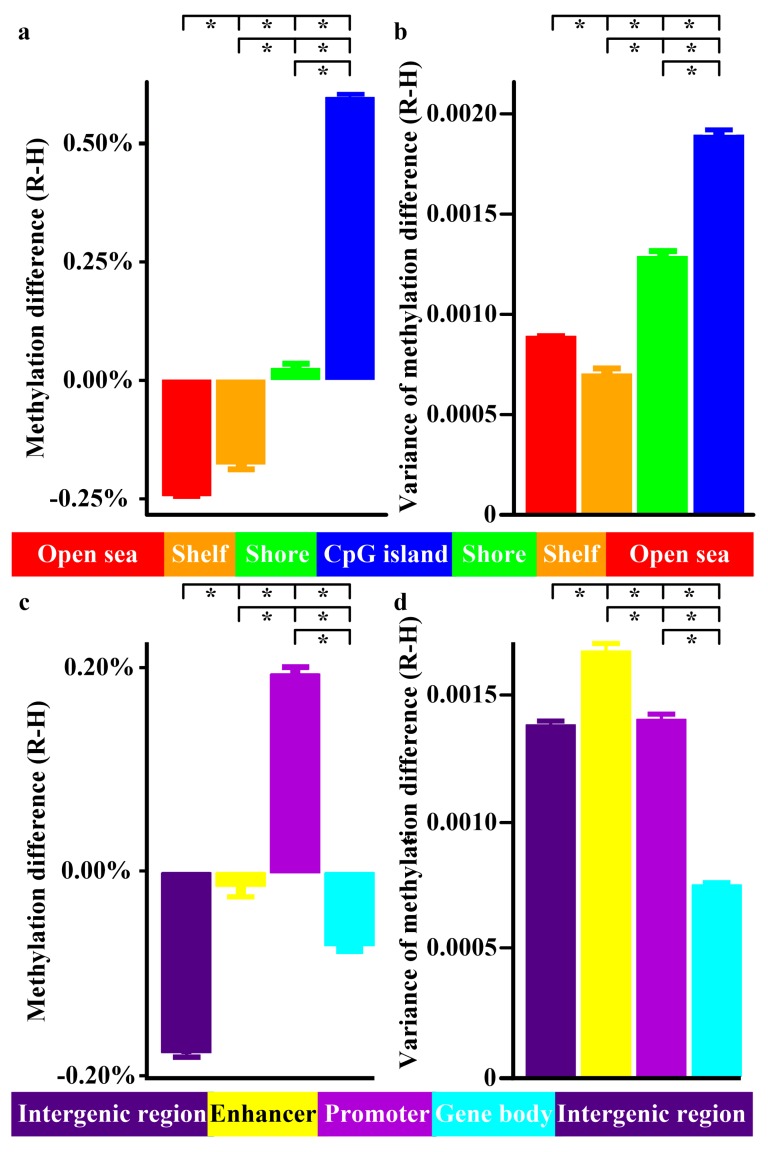
Methylation differences according to CpG features and genic characteristics. The bar charts showed the methylation difference (rheumatoid arthritis (R) minus healthy donor (H)) in CpG island, CpG shore, CpG shelf, open sea (**a**) and variance of methylation according to respective CpG features (**b**). Methylation difference in intergenic region, enhancer, promoter, gene body (**c**) and variance of methylation in respective genic characteristics (**d**) were also presented. * *p* < 0.001 for methylation difference and variance of methylation between different CpG features and genic characteristics.

Past studies suggest methylation alterations depended on CpG features [9]. However, whether similar phenomena existed in RA remained unexplored. Traditionally, CpG sites are classified into four classes according to their CpG features. CpG islands are genomic regions of >200 bp with a CG content of >50% and an observed/expected CpG ratio of >60%. CpG shores are located within 2 kb from CpG island). CpG shelves include regions 2–4 kb from CpG island. The remaining regions >4 kb from CpG island are defined as open seas [10]. To clarify whether methylation variations differed with respective CpG features, we classified CpG into CpG islands, CpG shores, CpG shelves, and open seas adopting similar classification schemes (Figure 1, Step 3). On average, CpG islands and CpG shores were hypermethylated in RA, and CpG shelves and open seas were hypomethylated in RA and methylation difference differed with respect to CpG features (Figure 2a). Overall, the methylation variance was most pronounced in CpG islands, CpG shores, followed by open seas and CpG shelves (*p* < 0.001) (Figure 2b).

### 3.4. Genic Characteristics Annotation

In addition to CpG features, evidence suggested methylation alterations differed with respect to genic characteristics [9]. To test these possibilities in RA, we annotated every CpG to enhancers, promoters, gene bodies, and intergenic regions (Figure 1, Step 4). Generally, CpG in promoters were hypermethylated and CpG in enhancers, gene bodies and intergenic regions were hypomethylated in RA, with significant methylation differences between different genic characteristics (Figure 2c). Furthermore, the methylation variance was most striking in enhancers, followed by promoters and intergenic regions, decreased in gene bodies (*p* < 0.001) (Figure 2d). When we further stratified CpG located in promoters according to their distance to transcription start sites, the results showed preferential methylation alterations near the transcription start sites (Figure S4).

### 3.5. Methylation Variation Linked to Transcription Variation

Since transcription is regulated through epigenetic marks, we subsequently set upon determining whether the presence of methylation alterations was linked to alterations in gene expression (Figure 1, Step 5). We divided CpG into high variance (methylation variance above mean methylation variance) and low variance (methylation variance below mean methylation variance). Enhancer CpG with high methylation variance was associated with greater variation in transcript abundance compared with enhancer CpG with low methylation variance (*p* < 0.001, Figure S5a,b). Promoter CpG with high methylation variance was associated with greater variation in transcript abundance compared with promoter CpG with low methylation variance (*p* < 0.001, Figure S5c,d). We next focused our analysis on CpG located in gene bodies. Again, a higher variance of gene expression was significantly associated with gene body CpG with higher methylation variance (*p* < 0.001, Supplementary Figure S5e,f).

### 3.6. Integration of Methylation and Expression Profiles

After confirming the association between methylation variation and expression variation, we interrogated methylation and expression profiles to identify differentially methylated genes and differentially expressed genes. We first identify genes with differentially-methylated regions (FDR < 0.05) (Figure 1, Step 6a). In the same time, differentially expressed genes (FDR < 0.05) were found (Figure 1, Step 6b). Since enhancer/promoter methylation was associated with decreased gene expression and gene body methylation was associated with increased gene expression [8,11], we intersected differentially methylated genes and differentially expressed genes to obtain genes with concomitant expression and methylation changes in enhancer/promoter/gene body (Step 7) for following analysis.

### 3.7. RA Genetically Associated Genes and Their Targets Preferentially Displaying Differential Methylation and Differential Expression

A growing body of literature suggested interaction of genetic loci and differentially methylated loci in phenotype determination [12]. To examine whether there was similar genetic–epigenetic interaction in RA, we utilized GWAS results on RA and non-RA traits and protein-protein interaction information from BioGRID to characterize genetic–epigenetic interaction in RA (Figure 1, Step 8; Figure S1). RA genetically associated genes and their interacting targets are more likely to exhibit differential methylation and differential expression than non-RA genetically associated genes and their interacting targets (Figure S6). This finding highlighted interaction of genetically associated genes and epigenetically associated genes in RA pathogenesis.

### 3.8. Ingenuity Pathway Analysis

To identify pathways and diseases associated with the differential methylation and differential expression in RA compared with healthy donors, we performed a pathway analysis using IPA. Dendritic cell maturation, inflammasome pathway, iNOS signaling, LPS/IL-1 mediated inhibition of RXR function, neuroinflammation signaling pathway, NF-κB signaling, PPAR signaling, Toll-like receptor signaling, TREM1 signaling and type 1 diabetes mellitus signaling were identified as enriched pathways (Figure S7, Table S3). Differentially methylated and differentially expressed genes were enriched for genes of atherosclerosis, atopic dermatitis, hematopoietic neoplasm, inflammation of joint, juvenile rheumatoid arthritis, polyarticular juvenile rheumatoid arthritis, rheumatic disease, rheumatoid arthritis, systemic autoimmune syndrome and viral infection as disease annotation (Figure S8, Table S4).

### 3.9. Upstream Regulator Deduction

**Figure 3:**
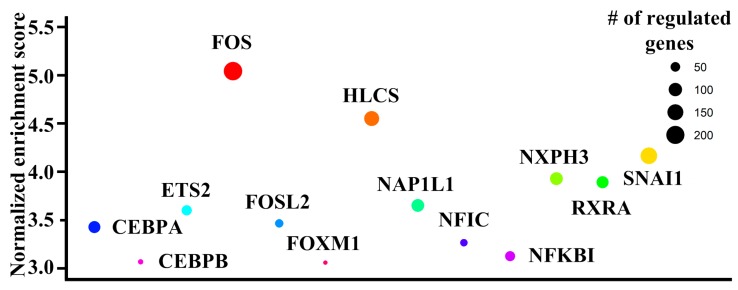
Transcription factors identified through iRegulon analysis. The bubble chart showed the transcription factors associated with differentially methylated and differentially expressed genes identified by iRegulon. Y-axis label represented normalized enrichment score. The sizes of the bubbles were proportional to the number of regulated genes with concomitant differential methylation and differential expression for each transcription factor.

Since altered DNA methylation in differentially methylated regions may contribute to transcriptional dysregulation through altered transcription factor binding [13], to gain insight into involved transcription factors, a network of transcription factors and their targets was constructed using iRegulon (Figure 1, Step 10). iRegulon revealed 13 transcription factors (CEBPA, CEBPB, ETS2, FOS, FOSL2, FOXM1, HLCS, NAP1L1, NFIC, NFKBI, NXPH3, RXRA, SNAI1) with significant enrichment of target genes in the network of genes with concomitant differential methylation and differential expression (Figure 3). These transcription factors had well-established roles in inflammation and immune cells development (Table S5).

### 3.10. Validation of Differential Methylation and Differential Expression in RA

**Figure 4:**
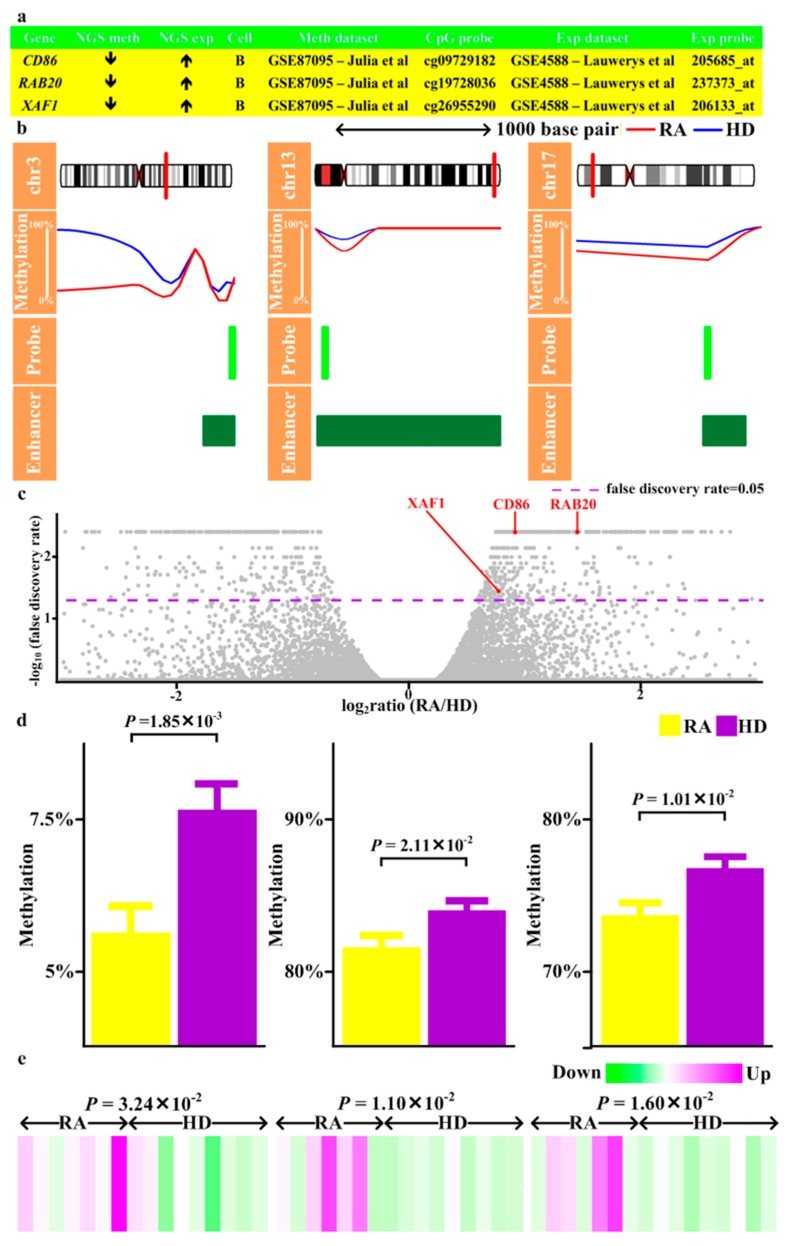
Validation of genes with differential methylation in enhancer and differential expression. (**a**) The results of methylation and expression obtained from next-generation sequencing (NGS meth, NGS exp), the cell subsets of validation dataset (Cell), the dataset of validation (Meth dataset, Exp dataset), and the probes of validation dataset (CpG probe, Exp probe). (**b**) Visualization of the methylation levels obtained from NGS in rheumatoid arthritis (RA) and healthy donors (HD) and location of validated CpG probe and enhancers. (**c**) Volcano plot of the −log_10_(false discovery rate) on the Y-axis versus expression change (log_2_ratio) on the X-axis. Of validated genes, (**d**) Methylation and (**e**) Expression levels of corresponding probes in the validation dataset.

**Figure 5:**
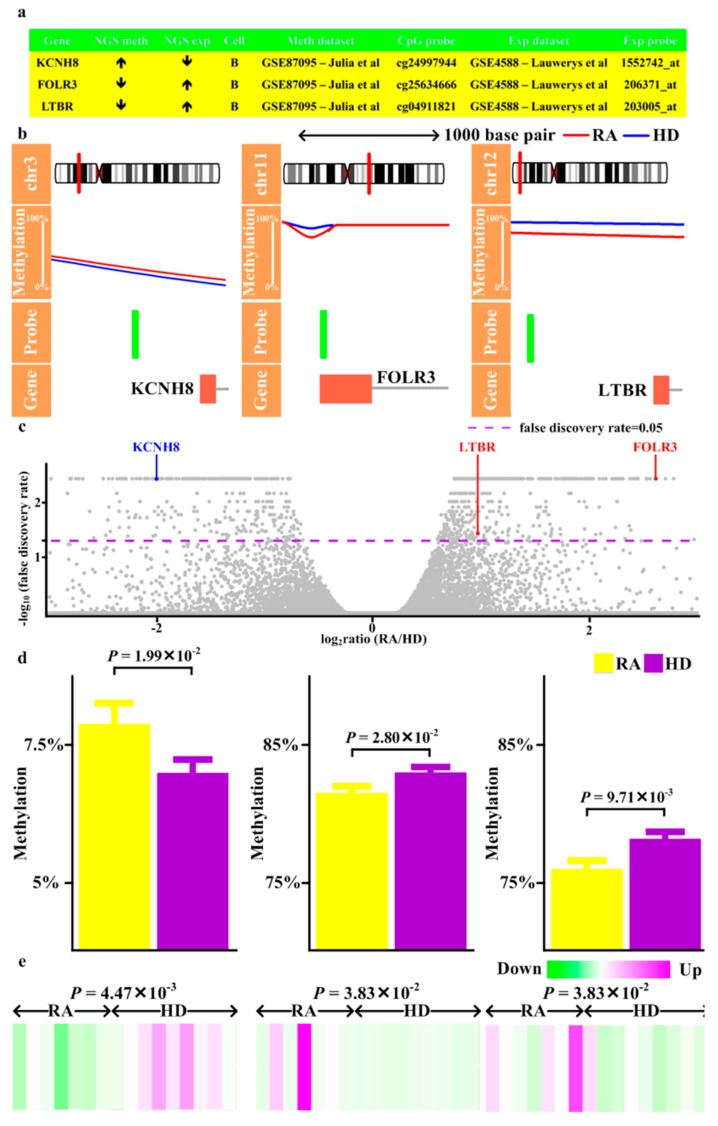
Validation of genes with differential methylation in promoter and differential expression. (**a**) The results of methylation and expression obtained from next-generation sequencing (NGS meth, NGS exp), the cell subsets of validation dataset (Cell), the dataset of validation (Meth dataset, Exp dataset), and the probes of validation dataset (CpG probe, Exp probe). (**b**) Visualization of the methylation levels obtained from NGS in RA and healthy donors (HD) and location of validated CpG probe superposed onto the genomic locations of genes. (**c**) Volcano plot of the -log_10_(false discovery rate) on the Y-axis versus expression change (log_2_ratio) on the X-axis. Of validated genes, (**d**) Methylation and (**e**) Expression levels of corresponding probes in the validation dataset.

**Figure 6:**
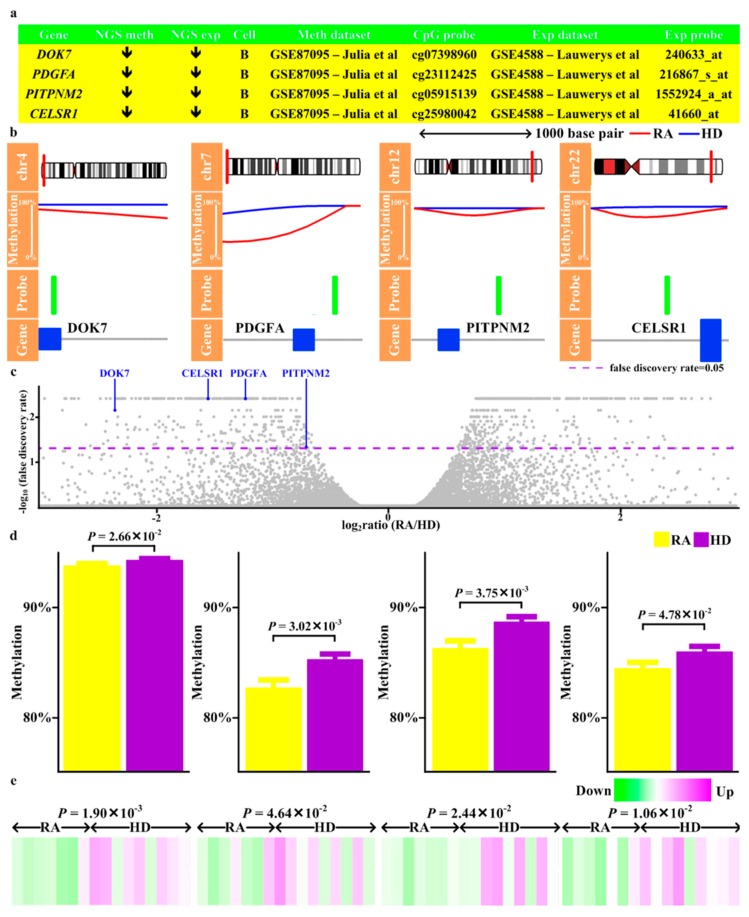
Validation of genes with differential methylation in gene body and differential expression. (**a**) The results of methylation and expression obtained from next-generation sequencing (NGS meth, NGS exp), the cell subsets of validation dataset (Cell), the dataset of validation (Meth dataset, Exp dataset), and the probes of validation dataset (CpG probe, Exp probe). (**b**) Visualization of the methylation levels obtained from NGS in RA and healthy donors (HD) and location of validated CpG probe superposed onto the genomic locations of genes. (**c**) Volcano plot of the -log_10_(false discovery rate) on the Y-axis versus expression change (log_2_ratio) on the X-axis. Of validated genes, (**d**) Methylation and (**e**) Expression levels of corresponding probes in the validation dataset.

To validate the results from next-generation sequencing, we retrieved previously reported methylation and expression patterns of RA CD4 T cells and B cells, both of which were major cellular subsets of PBMCs, from GEO (Figure 1, Step 11) (methylation and expression profiles of CD8 and monocyte unavailable). The magnitude of methylation aberrations across all validated genes was similar to previous studies [14] (Figure 4, Figure 5 and Figure 6). 10 genes with methylation alteration and expression deregulation were validated in B cells, including three (*CD86, RAB20, XAF1*) with enhancer hypomethylation and expression upregulation (Figure 4), one (*KCNH8*) with promoter hypermethylation and expression downregulation, two (*FOLR3, LTBR*) with promoter hypomethylation and expression upregulation (Figure 5), and four (*DOK7, PDGFA, PITPNM2, CELSR1*) with gene body hypomethylation and expression downregulation (Figure 6).

## 4. Discussion

Here we reported a comprehensive analysis of methylome and transcriptome in RA. By combining methyl-seq and RNA-seq data, this study provided a global map of the methylation profile in RA. Methylation alterations occurred across human genomes (Figure S3), varied with CpG features and genic characteristics (Figure 2) and associated with gene expression (Figure S5). RA genetically associated genes and their interacting targets preferentially displayed differential methylation and differential expression compared with non-RA genetically associated genes (Supplementary Figure S6). These methylation and transcription aberration associated with several autoimmune and infectious diseases (Figure S8, Table S4). Additionally, we identified several transcription factors as potential regulators (Figure 3). Moreover, 10 genes (*CD86, RAB20, XAF1, KCNH8, FOLR3, LTBR, DOK7, PDGFA, PITPNM2,* and *CELSR1*) with concomitant methylation and expression alterations in B cells were validated (Figure 4, Figure 5 and Figure 6). These results highlighted potential roles played by these genes in RA.

The scale of methylation differences across all validated genes was not large, similar to those reported in previous studies [14,15,16]. Studies suggested that traits-associated methylation changes were predominantly of small magnitude [16,17], tended to be subtle and long-lasting, with stronger but short-lived gene expression alterations [15]. Accumulating evidence further suggested functional consequences of such subtle methylation changes, with halving or doubling of gene transcription accompanying every 1% change in methylation [17]. These collectively supported the biological relevance of methylation alterations validated in this study.

Generally, PCA and HC based on the methylation levels revealed a clear phenotype-driven distinction between RA and healthy donors (Figure S2a,b), supportive of the potential of methylation as diagnostic marker. Similar conclusions were made in other autoimmune diseases, including SLE [18]. However, difference of methylation profiles between RA patients was also noted (Figure S2a), suggesting epigenetic heterogeneity of RA patients. Epigenetic alteration varies with different manifestations of autoimmune disease [18]. Evidence suggests genetic and clinical heterogeneity of RA [19], though it has not been fully defined and warrants further study. Since different serology status implied contrasting genetic architecture and transcriptome changes [20,21], it is tempting to speculate on the roles of autoantibodies in the difference of methylome, as one of patients was positive for anti-citrullinated protein antibodies (ACPA) and the other negative for ACPA. Large scales of studies combining clinical status, immunopathology, methylomics and transcriptomics analysis from ACPA+ vs ACPA- patients will provide valuable insight into the relationship between autoantibodies and epigenetic subsets of RA. This needed to be explored in future studies.

When we characterized methylation variation according to CpG features, CpG islands displayed the highest methylation variation compared with other CpG features (Figure 2b). Since CpG islands had the most pronounced correlation with gene expression level [22], this suggested that despite small methylation differences, there may be more biologically relevant regions of the genome.

In this study, we observed differential enhancer, promoter and gene body methylation between RA and healthy donors (Figure 2c). On average, promoter hypermethylation and enhancer and gene body hypomethylation were noted. Promoter hypermethylation has also been demonstrated in other autoimmune diseases, such psoriasis [23]. Interestingly, promoter hypermethylation was often correlated with gene downregulation [8]. These changes in promoter and gene body DNA methylation might be related to inadequate immune regulation [24] and exemplified by polycyclic forms of RA-asymptomatic during interepisodic period but flare-up intermittently [25].

With regards to methylation variation within promoters, increased methylation variation was noted in the vicinity of transcription start site (Figure S4). Since transcription factor binding sites were enriched in transcription start site [26], the presence of more dynamic DNA methylation in the vicinity of transcription start site provided higher flexibility for different transcription factor bindings under different conditions and thus transcription plasticity.

Our study highlighted that RA genetically associated genes and their interacting targets are more likely to exhibit differential methylation and differential expression than non-RA genetically associated genes (Figure S6). Although evidence of genetic–epigenetic interaction existed in past literature [12], interaction of genetically associated genes and epigenetically associated genes in autoimmune diseases such as RA was largely uncharacterized. Furthermore, interaction of genetically associated genes and epigenetically associated genes in RA raised the possibility of cooperation of these two interacting systems to facilitate gene regulation in RA pathogenesis. It is possible that both methylation and genetic alterations were necessary for RA development and altered DNA methylation may be a second hit contributing to penetrance as demonstrated by complex multifactorial traits [27] and supported by past studies of autoimmune diseases [28].

Pathway analysis of methylation and expression alterations suggested significantly multiple upregulated inflammatory pathway (including TREM1 signaling) and one downregulated pathway (PPAR signaling) (Figure S7). *TREM1* was expressed in monocyte and amplified production of IL-6 and TNF-α, both critical players in RA pathogenesis [29]. With regards to PPAR, PPAR activation downregulated NF-κB signaling, primed monocytes into anti-inflammatory properties, and exerted therapeutic effects on RA [30,31,32]. These findings support the importance of DNA methylation on the regulation of implicated pathways in RA.

In the diseasome analysis, genes with significantly different DNA methylation and expression alterations were associated with several diseases, including atherosclerosis, atopic dermatitis, hematopoietic neoplasm, inflammation of joint, juvenile rheumatoid arthritis, polyarticular juvenile rheumatoid arthritis, rheumatic disease, rheumatoid arthritis, systemic autoimmune syndrome and viral infection (Figure S8). Numerous reports linked RA with juvenile idiopathic arthritis, atopic dermatitis, atherosclerosis and lymphoma [33,34,35,36]. Viral infection also associated with RA in past epidemiology study [37]. However, it is previously unknown whether these diseases are also linked to RA epigenetically. This study was the first to suggest an epigenetic relationship between these diseases and RA.

When we applied iRegulon to decipher potential upstream regulator, several transcription factors were singled out (Figure 3). All these transcription factors were involved in various aspects of immunological responses (Table S5). Thus, it was conceivable that they participated in regulation of differentially methylated and differentially expressed genes in RA.

During validation with B cell microarray profiles, upregulated *CD86, RAB20, XAF1*, *FOLR3, LTBR* and downregulated *KCNH8*, *DOK7, PDGFA, PITPNM2, CELSR1* with corresponding methylation changes in enhancers/promoters/gene bodies were identified (Figure 4, Figure 5 and Figure 6). CD86 activated B cell proliferation and immunoglobulin secretion [38]. Moreover, *CD86* was increased in RA B cells and correlated with disease activity [39,40]. Thus, upregulated *CD86* may contribute to immune activation in RA (Figure S9). Considering *RAB20, RAB20* was upregulated by Crohn’s disease-associated polymorphism and vaccination and increases during B cell transformation (Table S3 of [41], Supplementary material S1 of [42,43]). It may be possible that increased *RAB20* contributes to B cell activation and facilitates RA development (Figure S9). Regarding *XAF1*, *XAF1* was one risk gene of sarcoidosis which implicated dysregulated immune responses [44]. Moreover, *XAF1* was downregulated during lymphocyte immortalization and sensitized lymphocyte to apoptosis [45,46]. As a result, *XAF1* has the potential to be involved in RA pathogenesis (Figure S9).

*KCNH8* was almost exclusively expressed in B cells [47] and *KCNH8* region was associated with susceptibility to autoimmune diseases, including Crohn’s disease and psoriasis [48]. However, its function in B cells remained unexplored. *FOLR3* was a member of folate receptor family. *FOLR3* was associated with hepatitis C virus clearance [49] and folate receptor-mediated STAT3 activation [50]. Therefore, upregulated *FOLR3* may contribute to immune activation (Figure S9). With regards to *LTBR*, *LTBR* activated NFkB and blockade of *LTBR* impaired humoral immune response and ameliorated arthritis in the animal model [51,52,53]. Thus, upregulated *LTBR* potentially activates humoral immunity and facilitated arthritis development (Figure S9).

*DOK7* belonged to a family of docking protein and *DOK7* inhibited malignant cell proliferation and increased leukemia patient survival [54,55]. *DOK7* downregulation may lead to increased B cell proliferation and aggravated RA (Figure S9). *PDGFA* was part of *PDGF* family and *PDGF* family members stimulated B cell growth [56]. Whether downregulated *PDGFA* represents one mechanism to counteract excessive inflammation is unknown (Figure S9). *PITPNM2* was implicated as a risk locus of multiple sclerosis [57] and allergic diseases [58] which were all linked to RA [34,59]. Furthermore, risk protective alleles of allergic disease and drug with anti-inflammatory effects in autoimmune diseases both increased *PITPNM2* expression (Supplementary Table 27 of [58,60]). As a result, decreased *PITPNM2* might enhance RA pathogenesis (Figure S9). *CELSR1* was part of the apoptosis network [61], inhibited proliferation of neural progenitor [62] and decreased in non-nodal mantle cell lymphoma [63]. Therefore, decreased *CELSR1* might facilitate B cell proliferation and therefore sustain immune responses in RA (Figure S9).

In this study, we detected methylation and transcription perturbations in *CD86*. Notably, abatacept, one approved treatment option for RA, decreased CD86 expression in B cells [64]. It was possible that genes with differential methylation and differential expression identified in this study hold therapeutic promises for RA in the future. These should be addressed by further studies.

Limitations of this work include the relatively small sample size due to the high cost of next-generation sequencing and failure to validate methylation results in CD4 T cells. This may be a result of potentially more aberrant methylation of B cells than CD4 T cells, as demonstrated in another autoimmune disease, Sjogren’s syndrome [65]. Future work was needed to fully characterize additional RA samples by next-generation sequencing with additional cell types.

In past decades, progress in understanding the molecular bases of disease pathogenesis and the application of new technologies greatly transformed our treatment of diseases [1]. Integration with multiomic data identified several novel genes and pathways as potential relevant therapeutic avenues that may be important dysregulated mediators at the interface of genetics, epigenetics, and RA pathogenesis. These results may be useful for the development of new, more effective biomarkers and therapeutics. With that goal in mind, future studies are necessary in order to characterize precisely the molecular mechanisms, the functional consequences, and the interactions between differential methylation and genetic risk factors in RA pathogenesis.

## Supplementary Materials

The following are available online at https://www.mdpi.com/2077-0383/8/9/1284/s1, Figure S1: Flowcharts of genetic-epigenetic interaction investigation, Figure S2: Principal component analysis and hierarchical clustering results, Figure S3: Circular representation of the methylome of rheumatoid arthritis peripheral blood mononuclear cells in different chromosomes, Figure S4: Distribution of methylation variance relative to transcription start sites in promoters, Figure S5: Transcriptional variance was associated with methylation variance at enhancers, promoters and gene bodies, Figure S6. Graphical representation of enrichment of RA genetically associated genes with differential methylation and differential expression or interacting with differentially methylated and differentially expressed genes, Figure S7: Top 10 significantly perturbed canonical pathways revealed by Ingenuity Pathway Analysis, Figure S8: Top 10 diseases associated with genes having concomitant differential methylation and differential expression in rheumatoid arthritis, Figure S9: Proposed mechanism of *CD86, RAB20, XAF1, FOLR3, LTBR, PDGFA, DOK7, PITPNM2, CELSR1* in B cells of rheumatoid arthritis, Table S1: Next-generation sequencing RNA quality of rheumatoid arthritis and healthy donor peripheral blood mononuclear cells, Table S2: HACER source cells utilized in enhancer annotation, Table S3: Top ten pathways identified by Ingenuity Pathway Analysis from differentially methylated and differentially expressed genes, Table S4: Top ten diseases identified by Ingenuity Pathway Analysis (IPA) from differentially methylated and differentially expressed genes, Table S5: Roles of identified transcription factors in immunity.
