# Supplementary material for: Next-Generation Sequencing Profiles of the Methylome and Transcriptome in Peripheral Blood Mononuclear Cells of Rheumatoid Arthritis
Source: J Clin Med. 2019 Aug 22;8(9):1284. doi: 10.3390/jcm8091284 (PMC6780767; doi:10.3390/jcm8091284)
Supplement: Supplementary file 1 [file jcm-08-01284-s001.pdf]

Journal of Clinical Medicine

Supplementary Materials and Methods

1. Study Participants

We recruited two patients with RA and two unrelated healthy donors without any autoimmune diseases from the same geographic area through advertisement. One RA patient was 64 years old female, negative for rheumatoid factor and anti-citrullinated protein antibodies (ACPA), erythrocyte sedimentation rate (ESR) 23 mm/h, C-reactive protein (CRP) 4.99 mg/dL, negative for joint erosions. The other RA patient was 64 years old female negative for joint erosions and rheumatoid factor, but had multiple poor prognosis factors, including positive for ACPA, ESR 112 mm/h, CRP 12.08 mg/dl [1]. All participants reported their ethnic group as Han Chinese. RA patients fulfilled the American College of Rheumatology (ACR) 1987 revised criteria and 2010 ACR/European League Against Rheumatism (EULAR) classification criteria for RA. The study was conducted in accordance with the Declaration of Helsinki and ethical approval was granted by the Kaohsiung Medical University Hospital institutional review board (KMUHIRB-G(II)-20180031).

2. DNA and RNA Extraction

Peripheral blood mononuclear cells (PBMCs) were separated by the Ficoll-Paque method, either for DNA extraction or for RNA isolation. DNA was extracted using a commercial kit (Geneaid, New Taipei, Taiwan). Total RNA was isolated by QIAmp RNA Blood Mini Kit (Qiagen, Hilden, Germany). The RNA quantity and quality were evaluated using the ND-1000 spectrophotometer (Nanodrop Technology, Wilmington, DE, USA) and the Agilent RNA 6000 labchip kit with Agilent 2100 Bioanalyzer instrument (Agilent Technologies, Santa Clara, CA, USA), respectively. The qualities of extracted RNA were shown in supplementary Table S1.

3. Methyl-Seq

For methylome profiling, TruSeq Methyl Capture EPIC protocol was adopted. Briefly, DNA was sonicated via ultrasonication to obtain products of 180–220 base pairs. DNA was then end-repaired, adenylate-tailed, ligated with methylated indexed-adapters to create pre-capture DNA libraries. The pre-capture libraries were hybridized to the EPIC oligos, purified by capture with Streptavidin beads and washed twice to remove nonspecific binding. After the last elution, hybridized products were subjected to bisulfite conversion with the reagent provided in the TruSeq Methyl Capture EPIC Library Prep Kit. The bisulfite-treated libraries were PCR-amplified for 11 cycles with Kapa HiFi HotStart Uracil + polymerase (Kapa Biosystems). Libraries were clustered on a V3 paired-end read flow cell and sequenced for 100 cycles (PE100) on an Illumina HiSeq 2500. Quality control of read sequences was performed using FastQC, removing reads with phred score < 30. FastQC was run on the trimmed sequences to verify quality control. Quality-trimmed paired-end reads were analyzed with MethylSeq v1.0, which employed Bismark (v0.12.2) for reads mapping and aligned to the reference human genome (hg19) using Bowtie2 (v2.2.2). Duplicated reads were removed. We used the surrogate variable analyses (SVA) method to capture unmeasured cellular effects and batch effects to remove interference from cellular composition [2-3]

4. RNA-Seq

Agilent's SureSelect Strand Specific RNA Library Preparation Kit was utilized for library construction. Briefly, RNA was first purified and fragmented employing poly-T oligo-attached magnetic beads followed by complementary DNA (cDNA) strand synthesis. Next, cDNA 3' ends were adenylated, and adapters ligated followed by library amplification. The libraries were size-selected with AMPure XP Beads (Beckman Coulter). The sequence was determined adopting sequencing-by-synthesis technology with the TruSeq SBS Kit. Raw sequences were obtained from the Illumina Pipeline software bcl2fastq v2.0 to generate 30 million reads per sample. The sequences

generated then went through a filtering process to obtain qualified reads. Trimmomatic was applied to trim or discard the reads based on the quality score. Qualified reads after filtering low-quality data were analyzed for gene expression estimation using TopHat/Cufflinks method. The gene expression level was normalized by calculating Fragments Per Kilobase of transcript per Million mapped reads (FPKM). We retrieved mRNA with FPKM > 0.3 since a threshold FPKM value of 0.3 balanced the numbers of false positives and false negatives [4]. SVA was utilized to adjust expression variation due to cell type and batch effects [5]. Statistical analysis was performed using Cuffdiff (Cufflinks version 2.2.1), with P value calculation for non-grouped samples using blind mode, in which all samples were treated as replicates of a single global condition and used to blind one model for a statistic test. Benjamini-Hochberg method for false discovery rate calculation was performed.

5. Principal Component Analysis (PCA) and Hierarchical Clustering (HC) of Methylation in RA and Healthy Donors

PCA is a technique that measures variations among samples within a multidimensional dataset and is useful at reducing the dimensionality of the dataset. PCA reduces the high dimensionality of a dataset by transforming the large number of regions to a few principal components. A given principal component (PC) describes a specific pattern of DNA methylation across samples. Each sample in the dataset is assigned a score for each principal component, indicating the relative contribution of each PC-related pattern to the sample's overall variation. Each PC is independent from the others and accounts for a particular amount of variance within the dataset. The principal components are ordered so that the first few include most of the variation present in the original data and are used to emphasize grouping structure in the data. A plot of the first two principal components reveals a biologically meaningful clustering of the samples. MethylKit was applied for PCA to assess relevance of differential methylation to RA [6]. Following the PCA, HC analysis employing Ward's clustering method with squared Euclidean distances was performed to assess similarities in methylation and to identify clusters of samples (Figure 1, Step 1).

6. OmicCircos Visualization

To explore whether the methylation pattern changed with regards to chromosome location and chromosome number, the methyl-seq data were first categorized according to their respective chromosome number and then ordered by their location in respective chromosome. The methylation difference of RA and healthy donors in every sequenced CpG site was calculated by subtracting mean methylation level of healthy donors from mean methylation level of RA. For a visual representation of methylation level of RA, methylation level of healthy donors, and methylation difference for every sequenced CpG sites, Omiccircos was employed (Figure 1, Step 2) [7].

7. CpG Features Mapping

To map CpG sites to CpG features, CpG islands ((1) GC content above 50%, (2) ratio of observed-to-expected number of CpG dinucleotides above 0.6, and (3) length greater than 200 base pairs), CpG shores (2 kilobase upstream/downstream from the ends of the CpG islands), CpG shelf (2-4 kilobase upstream/downstream of the CpG islands), and open seas (otherwise) were identified [8]. Mapping of CpG sites to CpG features was provided by the annotatr package (Figure 1, Step 3) [9]. Differences between groups were assessed with analysis of variance (ANOVA) and post hoc Tukey's test.

8. Genic Characteristics Annotation

To identify enhancers, promoters, gene bodies and intergenic regions, we utilized HACER and ChIPpeakAnno [10-11]. HACER integrated ENCODE transcription factor ChIP-seq, FANTOM5 Cap Analysis of Gene Expression (CAGE) profiles, GRO-seq and PRO-seq dataset, expression quantitative trait locus (eQTL) analysis and 4DGenome chromatin interaction studies to catalogue

and annotate cell-type-specific enhancers [10]. Genomic coordinates of enhancer regions in 38 PBMC cells (Supplementary Table S2) were retrieved from HACER [10]. Genomic coordinates of promoters (region from -2500 to 500 bp relative to the transcription start site) [12], gene body (other transcribed region), and intergenic regions (otherwise) were recorded with ChIPpeakAnno suite of programs [11]. CpG sites were annotated to enhancers, promoters, gene bodies, and intergenic regions accordingly (Figure 1, Step 4). Methylation differences between different genic characteristics were evaluated using ANOVA with post hoc Tukey's test.

9. Methylation-Expression Correlation

To explore the correlation between methylation and expression, we integrated methyl-seq and RNA-seq results (Figure 1, Step 5). The variance of methylation and variance of gene expression (FPKM $\log_2$ ratio) was calculated for all CpG sites and genes, similar to previous protocols [13]. CpG sites with methylation variance above mean methylation variance were categorized as CpG sites with high methylation variance. CpG sites with methylation variance below mean methylation variance were categorized as CpG sites with low methylation variance. The variance of gene expression between CpG sites with high methylation variance and CpG sites with low methylation variance was compared by Student's t test.

10. Identification of Differentially Methylated Region and Annotation of Genes with Differential Methylation and Differential Expression

The mapped reads of methyl-seq were used as input in methylKit for further analysis [6]. To identify extended differentially methylated region, logistic regression test was applied to compare fraction of methylated CpG sites across RA and healthy donor groups in differentially methylated regions. Following the differential methylation test and calculation of P values, Benjamini-Hochberg method was implemented to derive false discovery rate for P value correction. Regions with false discovery rate less than 0.05 were defined as significantly differentially methylated (Figure 1, Step 6a). GenomicRanges was used to map identified differentially methylated region annotation to respective genes. Similarly, genes with expression false discovery rate less than 0.05 were defined as the significantly differentially expressed (Figure 1, Step 6b).

11. Integration of Methylome and Transcriptome Data

Since enhancer and promoter methylation correlated with decreased gene expression and gene body methylation was associated with increased gene expression [14-15], we identified upregulated genes in RNA-seq with enhancer/promoter hypomethylation or gene body hypermethylation in methyl-seq and downregulated genes in RNA-seq with enhancer/promoter hypermethylation or gene body hypomethylation in methyl-seq (Figure 1 Step 7). Genes with concomitant differential methylation and differential expression were entered as input for following analysis.

12. Genetic-Epigenetic Interaction Investigation

To explore the interaction between the genes with concomitant differential methylation and differential expression and the RA genetically associated genes, we integrated past genome-wide association study (GWAS) results and protein-protein interaction information from BioGRID, similar to previous approaches [16-17] (Figure 1 Step 8, supplementary Figure S1). In brief, a snapshot of the GWAS catalog was downloaded on 28 December 2018 from the NHGRI (https://www.ebi.ac.uk/gwas). For each reported single nucleotide polymorphism (SNP), SNPs in linkage disequilibrium (LD) were defined based on 1000 genomes Project Consortium phase 1 data as those with $r^2 \geq 0.8$, using HaploReg [17]. The SNPs associated with RA or in LD with SNPs associated with RA were considered RA-associated loci. Traits-associated loci were all SNPs and SNPs in LD with SNPs for any disease/trait in GWAS catalog. RA-associated loci and traits-associated loci were annotated to respective genes to obtain RA genetically associated genes and traits genetically associated genes with the aid of FUMA [18]. FUMA was an integrative

web-based platform accommodating information from multiple biological resources to facilitate positional and expression quantitative trait loci (eQTL) mapping to provides gene-based, pathway and tissue enrichment results. SNPs are subsequently mapped to genes based on integration of positional context and functional consequences on genes. NonRA genetically associated genes were obtained by excluding RA genetically associated genes from traits genetically associated genes. Interaction targets of RA genetically associated genes and nonRA genetically associated genes were curated by BioGRID. The enrichment of RA genetically associated genes with differential methylation and differential expression or targeting genes with differential methylation and differential expression was evaluated with Chi-square test [17].

13. Ingenuity Pathway Analysis

To clarify the function of genes with differential methylation and differential expression, Ingenuity Pathway Analysis (Qiagen) was applied to annotate the most over-represented canonical pathways and related diseases (Figure 1 Step 9) by submitting the gene lists as defined above. P values less than 0.05 were considered significant.

14. Upstream Regulator Deduction

The iRegulon plugin was used to reveal candidate transcription factors regulating genes with differential methylation and differential expression in RA PBMCs (Figure 1 Step 10) [19]. iRegulon utilized cis-regulatory sequence analysis to reverse-engineer the transcriptional regulatory network underlying a co-expressed gene set. It integrated the transcription factor information from various databases including Transfac, Jaspar, Encode, Swissregulon and Homer, and detected enriched transcription factor motifs and optimal sets of their direct targets by means of genome-wide ranking and recovery. The corresponding Normalized Enrichment Score (NES) was obtained from iRegulon. The higher the scores were, the more reliable the results were.

14. Validation of Genes with Concomitant Differential Methylation and Differential Expression

PBMCs contained multiple cellular subsets, including CD4 T cells, CD8 T cells, and B cells and monocytes. To confirm differential methylation of enhancer/promoter/gene body and differential expression of genes between RA and healthy donors, we searched existing literature and GEO database for methylation and transcription profiles of CD4 T cells, CD8 T cells, B cells and monocytes in RA and healthy donors. Only CD4 T cells and B cells had concurrent dataset of methylation and expression. Thus, we retrieved methylation and expression profiles of CD4 cells (GSE71841, GSE4588) and methylation and expression profiles of B cells (GSE87095, GSE4588) in RA and healthy donors (Figure 1, Step 11). Methylation and expression profiles obtained from GEO dataset were analyzed with GEO2R [20]. P values less than 0.05 were considered statistically significant.

Reference

1. Smolen, J.S.; Landewé, R.; Bijlsma, J.; Burmester, G.; Chatzidionysiou, K.; Dougados, M.; Nam, J.; Ramiro, S.; Voshaar, M.; van Vollenhoven, R.; et al. EULAR recommendations for the management of rheumatoid arthritis with synthetic and biological disease-modifying antirheumatic drugs: 2016 update. *Ann Rheum Dis.* **2017**, *76*, 960–977.
2. Li, J.; Zhu, X.; Yu, K.; Jiang, H.; Zhang, Y.; Deng, S.; Cheng, L.; Liu, X.; Zhong, J.; Zhang, X.; et al. Genome-Wide Analysis of DNA Methylation and Acute Coronary Syndrome. *Circ Res.* **2017**, *120*, 1754–1767.
3. Kaushal, A.; Zhang, H.; Karmaus, W.J.J.; Ray, M.; Torres, M.A.; Smith, A.K.; Wang, S.L. Comparison of different cell type correction methods for genome-scale epigenetics studies. *Bmc Bioinform.* . **2017**, *18*, 216.
4. Hart, T.; Komori, H.K.; LaMere, S.; Podshivalova, K.; Salomon, D.R. Finding the active genes in deep RNA-seq gene expression studies. *Bmc Genom.* . **2013**, *14*, 778.

5. Leek, J.T.; Storey, J.D. Capturing heterogeneity in gene expression studies by surrogate variable analysis. *Plos Genet.* **2007**, *3*, 1724–1735.
6. Akalin, A.; Kormaksson, M.; Li, S.; Garrett-Bakelman, F.E.; Figueroa, M.E.; Melnick, A.; Mason, C.E. methylKit: a comprehensive R package for the analysis of genome-wide DNA methylation profiles. *Genome Biol.* **2012,** *13*, R87.
7. Hu, Y.; Yan, C.; Hsu, C.H.; Chen, Q.R.; Niu, K.; Komatsoulis, G.A.; Meerzaman, D. OmicCircos: A Simple-to-Use R Package for the Circular Visualization of Multidimensional Omics Data. *Cancer Inf.* . **2014**, *13*, 13–20.
8. Weber, A.; Schwarz, S.C.; Tost, J.; Trümbach, D.; Winter, P.; Busato, F.; Tacik, P.; Windhorst, A.C.; Fagny, M.; Arzberger, T.; et al. Epigenome-wide DNA methylation profiling in Progressive Supranuclear Palsy reveals major changes at DLX1. *Nat Commun.* **2018**, *9*, 2929.
9. Cavalcante, R.G.; Sartor, M.A. annotatr: genomic regions in context. *Bioinform.* . **2017**, *33*, 2381–2383.
10. Wang, J.; Dai, X.; Berry, L.D.; Cogan, J.D.; Liu, Q.; Shyr, Y. HACER: an atlas of human active enhancers to interpret regulatory variants. *Nucleic Acids Res.* **2019,** *47*, D106–D112.
11. Zhu, L.J.; Gazin, C.; Lawson, N.D.; Pagès, H.; Lin, S.M.; Lapointe, D.S.; Green, M.R. ChIPpeakAnno: a Bioconductor package to annotate ChIP-seq and ChIP-chip data. BMC *Bioinform.* . **2010**, *11*, 237.
12. FitzGerald, P.C.; Shlyakhtenko, A.; Mir, A.A.; Vinson, C. Clustering of DNA sequences in human promoters. *Genome Res.* **2004**, *14*, 1562–1574.
13. Li, S.; Garrett-Bakelman, F.E.; Chung, S.S.; Sanders, M.A.; Hricik, T.; Rapaport, F.; Patel, J.; Dillon, R.; Vijay, P.; Brown, A.L.; et al. Distinct evolution and dynamics of epigenetic and genetic heterogeneity in acute myeloid leukemia. *Nat Med.* **2016**, *22*, 792–799.
14. Maunakea, A.K.; Nagarajan, R.P.; Bilenky, M.; Ballinger, T.J.; D'Souza, C.; Fouse, S.D.; Johnson, B.E.; Hong, C.; Nielsen, C.; Zhao, Y.; et al. Conserved role of intragenic DNA methylation in regulating alternative promoters. *Nat.* **2010**, *466*, 253–257.
15. Aran, D.; Sabato, S.; Hellman, A. DNA methylation of distal regulatory sites characterizes dysregulation of cancer genes. *Genome Biol.* **2013,** *14*, R21.
16. Oughtred, R.; Stark, C.; Breitkreutz, B.J.; Rust, J.; Boucher, L.; Chang, C.; Kolas, N.; O'Donnell, L.; Leung, G.; McAdam, R.; et al. The BioGRID interaction database: 2019 update. *Nucleic Acids Res.* **2019,** *47***,** D529–D541.
17. Walsh, A.M.; Whitaker, J.W.; Huang, C.C.; Cherkas, Y.; Lamberth SL, Brodmerkel C, Curran, M.E.; Dobrin, R. Integrative genomic deconvolution of rheumatoid arthritis GWAS loci into gene and cell type associations. *Genome Biol.* **2016**, *17*, 79.
18. Watanabe, K.; Taskesen, E.; van Bochoven, A.; Posthuma, D. Functional mapping and annotation of genetic associations with FUMA. *Nat Commun.* **2017**, *8*, 1826.
19. Verfaillie, A.; Imrichova, H.; Janky, R.; Aerts, S. iRegulon and i-cisTarget: Reconstructing Regulatory Networks Using Motif and Track Enrichment. *Curr Protoc Bioinform.* **2015**, *52*, 2.16.1–39.
20. Barrett, T.; Wilhite, S.E.; Ledoux, P.; Evangelista, C.; Kim, I.F.; Tomashevsky, M.; Marshall, K.A.; Phillippy, K.H.; Sherman, P.M.; Holko, M.; et al. NCBI GEO: archive for functional genomics data sets--update. *Nucleic Acids Res.* **2013,** *41*, D991–D995.

Table S1. Next-generation sequencing RNA quality of rheumatoid arthritis and healthy donor peripheral blood mononuclear cells.

	**OD_{260}/OD_{280}**	**RIN**
RA1-PBMC	1.97	9.7
RA2-PBMC	1.96	9.5
HD1-PBMC	1.96	9.4
HD2-PBMC	1.99	9.8

RA1-PBMC, RA2-PBMC: Rheumatoid arthritis peripheral blood mononuclear cells; HD1-PBMC, HD2-PBMC: Healthy donor peripheral blood mononuclear cells; OD: Optical Density; RIN: RNA integrity number.

Table S2. HACER source cells utilized in enhancer annotation.

ARH-77	ATN-1	BALL-1	CD4
CTB-1	Daudi	DAUDI	DS-1
GM12004	GM12750	GM12878	GM12878 ENCODE
HD-Mar2	HPB-ALL	HuT 102 TIB-162	Jurkat
KHYG-1	Ki-JK	Ly-1	MLMA
Mo	MV4-11	MV-4-11	Nalm6
NALM-6	PCM6	P30	P31
RAJI	REH	RPMI1788	SKW-3
SLVL	THP-1	U936	U-937 DE-4
WIL2-NS	XPL 17		

Table S3. Top ten pathways identified by Ingenuity Pathway Analysis (IPA) from differentially methylated and differentially expressed genes.

Pathway	Genes with differential methylation and differential expression
Dendritic cell maturation	*ICAM1, MYD88, HLA-A, NFKBIE, TLR2, COL5A3, NFKBIA, IL1RN, FSCN1, HLA-DMB, FCER1G, IL1B, CD86, IRS2, LTBR, TNFRSF1B*
Inflammasome pathway	*MYD88, PYCARD, CTSB, IL1B, P2RX7*
iNOS signaling	*FOS, NFKBIA, MYD88, NFKBIE, IFNGR2, IRAK3*
LPS/IL-1 mediated inhibition of RXR function	*MYD88, CHST15, SMOX, IL1RN, SULT1A1, IL1B, ALDH3B1, CHST13, TNFRSF1B, RXRA, ABCC4, MGST3, ACSL1*
Neuroinflammation signaling pathway	*CD200, ICAM1, HLA-A, PYCARD, MYD88, TLR8, IFNGR2, FZD1, IRAK3, FAS, TLR2, HMOX1, FOS, HLA-DMB, IL1B, CD86, IRS2, P2RX7*
NF-κB signaling	*MYD88, NFKBIE, FLT4, TLR8, IRAK3, TLR2, NFKBIA, IL1RN, FCER1G, IL1B, IRS2, INSR, LTBR, TRAF5, TNFRSF1B, PDGFRB*
PPAR signaling	*FOS, NFKBIA, IL1RN, PDGFA, NFKBIE, IL1B, INSR, TNFRSF1B, RXRA, PDGFRB*
Toll-like receptor signaling	*TLR2, FOS, NFKBIA, MYD88, IL1RN, TLR8, IL1B, IRAK3*
TREM1 signaling	*TLR2, TREM1, ICAM1, MYD88, TLR8, IL1B, CD86*
Type I diabetes mellitus signaling	*SOCS3, NFKBIA, HLA-A, MYD88, NFKBIE, HLA-DMB, IFNGR2, FCER1G, IL1B, CD86, TNFRSF1B, FAS*

Table S4. Top ten diseases identified by Ingenuity Pathway Analysis (IPA) from differentially methylated and differentially expressed genes.

Disease	Genes with differential methylation and differential expression
Atherosclerosis	*FOS, FOSB, JUNB, KLF4, KLF6, ZFP36*
Atopic dermatitis	*CXCR4, DUSP1, FAM153A/FAM153B, FCER1G, FCER2, IFI30, JUNB, LGALS1, NR4A2, TNFSF10, ZFP36*
Hematopoietic neoplasm	*CD86, CEBPA, CELSR1, FAS, HMOX1, KIT, PDGFRB, TNFRSF8*
Inflammation of joint	*ADM, BLK, CCR1, CD72, CEBPB, CXCR4, DUSP1, FOS, FOSB, G0S2, GADD45B, HIST1H2AC, IL1B, KLF13, LGALS1, NFKBIA, NR4A1, NR4A2, PLAUR, THBS1, TLR2, VASH1, ZFP36, ZNF281*
Juvenile rheumatoid arthritis	*ADM, CCR1, CEBPB, FOS, FOSB, GADD45B, IL1B, LGALS1, NFKBIA, NR4A1, NR4A2, PLAUR, THBS1, VASH1, ZFP36, ZNF281*
Polyarticular juvenile rheumatoid arthritis	*ADM, CCR1, CEBPB, FOS, FOSB, GADD45B, IL1B, LGALS1, NFKBIA, NR4A1, NR4A2, PLAUR, THBS1, ZFP36*
Rheumatic disease	*ADM, BLK, BST2, CCR1, CD72, CEBPB, CXCR4, DUSP1, FOS, FOSB, G0S2, GADD45B, HIST1H2AC, IL1B, KLF13, LGALS1, MX1, NFKBIA, NR4A1, NR4A2, PLAUR, SLAMF7, THBS1, TLR2, TYMP, USP18, VASH1, ZFP36, ZNF281*
Rheumatoid arthritis	*ADM, CCR1, CEBPB, CXCR4, DUSP1, FOS, FOSB, G0S2, GADD45B, HIST1H2AC, IL1B, KLF13, LGALS1, NFKBIA, NR4A1, NR4A2, PLAUR, THBS1, TLR2, VASH1, ZFP36, ZNF281*
Systemic autoimmune syndrome	*ADM, BST2, CCR1, CEBPB, CXCR4, DUSP1, FAS, FOS, FOSB, G0S2, GADD45B, HIST1H2AC, IL1B, KLF13, LGALS1, MX1, NFKBIA, NR4A1, NR4A2, PLAUR, SLAMF7, THBS1, TLR2, TYMP, USP18, VASH1, ZFP36, ZNF281*
Viral infection	*ACSL1, CD86, CEBPD, CTSB, CXCR4, FAS, HIST1H2AC, ICAM1, IL1B, IRS2, NFIL3, NFKBIA, P2RX7, SIGLEC1, SLC2A3, TIMP2, TLR2, TNFSF10*

Table S5. Roles of identified transcription factors in immunity.

	Roles in immunity	Reference
CEBPA	Regulates IFNγ expression in T cells	[1]
CEBPB	Regulates differentiation of dendritic cells and macrophage and inhibits monocyte apoptosis	[2-3]
ETS2	Induces miR-155 which facilitates inflammation	[4]
FOS	Regulator of IFNγ and TNF-α	[5]
FOSL2	Directs Th17 cell development	[6]
FOXM1	Induces NF-κB and JAK/STAT signaling, increases TNF-α and IL-6 production	[7-8]
HLCS	Deficiency causes immunological and inflammatory disorder	[9]
NAP1L1	Higher expression in lymphoma and involved in DNA replication in B cells	[10-11]
NFIC	Modulates B cell number, IL-6, IL-8, and TNF-α expression	[12-13]
NFKB1	Mutation causes immunodeficiency and autoimmune deficiency	[14]
NXPH3	One of monocyte associated genes, up-regulated in macrophages phagocytizing infected cells	[Supplementary Table S5 of 15, Supplementary Table S2 of 16]
RXRA	Alters T-lymphocyte proliferation and apoptosis	[17]
SNAI1	Also called Snail, expressed in T cells and B cells, stimulates TNF-α and IL-6 production	[18-21]

References (cont.)

21. Tanaka, S.; Tanaka, K.; Magnusson, F.; Chung, Y.; Martinez, G.J.; Wang, Y.H.; Nurieva, R.; Kurosaki, T.; Dong, C. CCAAT/enhancer-binding protein α negatively regulates IFN-γ expression in T cells. *J Immunol.* **2014**, *93*, 6152–6160.
22. Zhou, H.; Xiao, J.; Wu, N.; Liu, C.; Xu, J.; Liu, F.; Wu, L. MicroRNA-223 Regulates the Differentiation and Function of Intestinal Dendritic Cells and Macrophages by Targeting C/EBPβ. *Cell Rep.* **2015**, *13*, 1149–1160.
23. Tamura, A.; Hirai, H.; Yokota, A.; Sato, A.; Shoji, T.; Kashiwagi T. ; Iwasa, M.; Fujishiro, A.; Miura, Y.; Maekawa, T. Accelerated apoptosis of peripheral blood monocytes in Cebpb-deficient mice. *Biochem Biophys Res Commun.* **2015**, *464*, 654–658.
24. Quinn, S.R.; Mangan, N.E.; Caffrey, B.E.; Gantier, M.P.; Williams, B.R.; Hertzog, P.J.; McCoy, C.E.; O'Neill, L.A. The role of Ets2 transcription factor in the induction of microRNA-155 (miR-155) by lipopolysaccharide and its targeting by interleukin-10. *J Biol Chem.* **2014**, *289*, 4316–4325.
25. VanDenBerg, K.R.; Freeborn, R.A.; Liu, S.; Kennedy, R.C.; Zagorski, J.W.; Rockwell, C.E. Inhibition of early T cell cytokine production by arsenic trioxide occurs independently of Nrf2. *Plos One.* **2017,** 12, e0185579.
26. Ciofani, M.; Madar, A.; Galan, C.; Sellars, M.; Mace, K.; Pauli, F.; Agarwal, A.; Huang, W.; Parkhurst, C.N.; Muratet, M.; et al. A validated regulatory network for Th17 cell specification. *Cell.* **2012**, *151*, 289–303.
27. Zhu-Ge, D.; Yang, Y.P.; Jiang, Z.J. Knockdown CRNDE alleviates LPS-induced inflammation injury via FOXM1 in WI-38 cells. *Biomed Pharm.* **2018**, *103*, 1678–1687.
28. Zeng, R.M.; Lu, X.H.; Lin, J.; Hu, J.; Rong, Z.J.; Xu, W.C.; Liu, Z.W.; Zeng, W.T. Knockdown of FOXM1 attenuates inflammatory response in human osteoarthritis chondrocytes. *Int Immunopharmacol.* **2019**, *68*, 74–80.
29. Kuroishi, T. Regulation of immunological and inflammatory functions by biotin. *Can J Physiol Pharm.* **2015**, *93*, 1091–1096.
30. Wu, C.H.; Sahoo, D.; Arvanitis, C.; Bradon, N.; Dill, D.L.; Felsher, D.W. Combined analysis of murine and human microarrays and ChIP analysis reveals genes associated with the ability of MYC to maintain tumorigenesis. *Plos Genet.* **2008,** 4, e1000090.
31. Gupta, N.; Thakker, S.; Verma, S.C. KSHV encoded LANA recruits Nucleosome Assembly Protein NAP1L1 for regulating viral DNA replication and transcription. *Sci. Rep.* **2016**, *6*, 32633.
32. Wahlestedt, M.; Ladopoulos, V.; Hidalgo, I.; Sanchez Castillo, M.; Hannah, R.; Säwén, P.; Wan, H.; Dudenhöffer-Pfeifer, M.; Magnusson, M.; Norddahl, G.L.; et al. Critical Modulation of Hematopoietic Lineage Fate by Hepatic Leukemia Factor. *Cell Rep.* **2017**, *21*, 2251–2263.
33. Zhang, J.; Zhang, Y.; Lv, H.; Yu, Q.; Zhou, Z.; Zhu, Q.; Wang, Z.; Cooper, P.R.; Smith, A.J.; Niu, Z.; et al. Human stem cells from the apical papilla response to bacterial lipopolysaccharide exposure and anti-inflammatory effects of nuclear factor I C. *J Endod.* **2013**, *39*, 1416–1422.
34. Tuijnenburg, P.; Lango Allen, H.; Burns, S.O.; Greene, D.; Jansen, M.H.; Staples, E.; Stephens, J.; Carss, K.J.; Biasci, D.; Baxendale, H.; et al. Loss-of-function nuclear factor κB subunit 1 (NFKB1) variants are the most common monogenic cause of common variable immunodeficiency in Europeans. *J Allergy Clin Immunol.* **2018**, *142*, 1285–1296.
35. Waddell, S.J.; Popper, S.J.; Rubins, K.H.; Griffiths, M.J.; Brown, P.O.; Levin, M.; Relman, D.A. Dissecting interferon-induced transcriptional programs in human peripheral blood cells. *Plos One.* **2010,** 5, e9753.
36. Terkawi, M.A.; Takano, R.; Furukawa, A.; Murakoshi, F.; Kato, K. Involvement of β-defensin 130 (DEFB130) in the macrophage microbicidal mechanisms for killing Plasmodium falciparum. *Sci Rep.* **2017**, *7*, 41772.
37. Stephensen, C.B.; Borowsky, A.D.; Lloyd, K.C. Disruption of Rxra gene in thymocytes and T lymphocytes modestly alters lymphocyte frequencies, proliferation, survival and T helper type 1/type 2 balance. *Immunol. .* **2017**, *121*, 484–498.
38. Marcuello, M.; Mayol, X.; Felipe-Fumero, E.; Costa, J.; López-Hierro, L.; Salvans, S.; Alonso, S.; Pascual, M.; Grande, L.; Pera, M. Modulation of the colon cancer cell phenotype by pro-inflammatory macrophages: A preclinical model of surgery-associated inflammation and tumor recurrence. *Plos One.* **2018,** 13, e0192958.
39. Pioli, P.D.; Dahlem, T.J.; Weis, J.J.; Weis, J.H. Deletion of Snai2 and Snai3 results in impaired physical development compounded by lymphocyte deficiency. *Plos One.* **2013,** 8, e69216.
40. Hsu, D.S.; Wang, H.J.; Tai, S.K.; Chou, C.H.; Hsieh, C.H.; Chiu, P.H.; Chen, N.J.; Yang, M.H. Acetylation of snail modulates the cytokinome of cancer cells to enhance the recruitment of macrophages. *Cancer Cell.* **2014**, *226*, 534–548.

41. Chen, S.Y.; Shiau, A.L.; Li, Y.T.; Lin, C.C.; Jou, I.M.; Liu, M.F.; Wu, C.L.; Wang, C.R. Transcription factor snail regulates tumor necrosis factor α-mediated synovial fibroblast activation in the rheumatoid joint. *Arthritis. Rheumatol.* **2015**, *67*, 39–50.

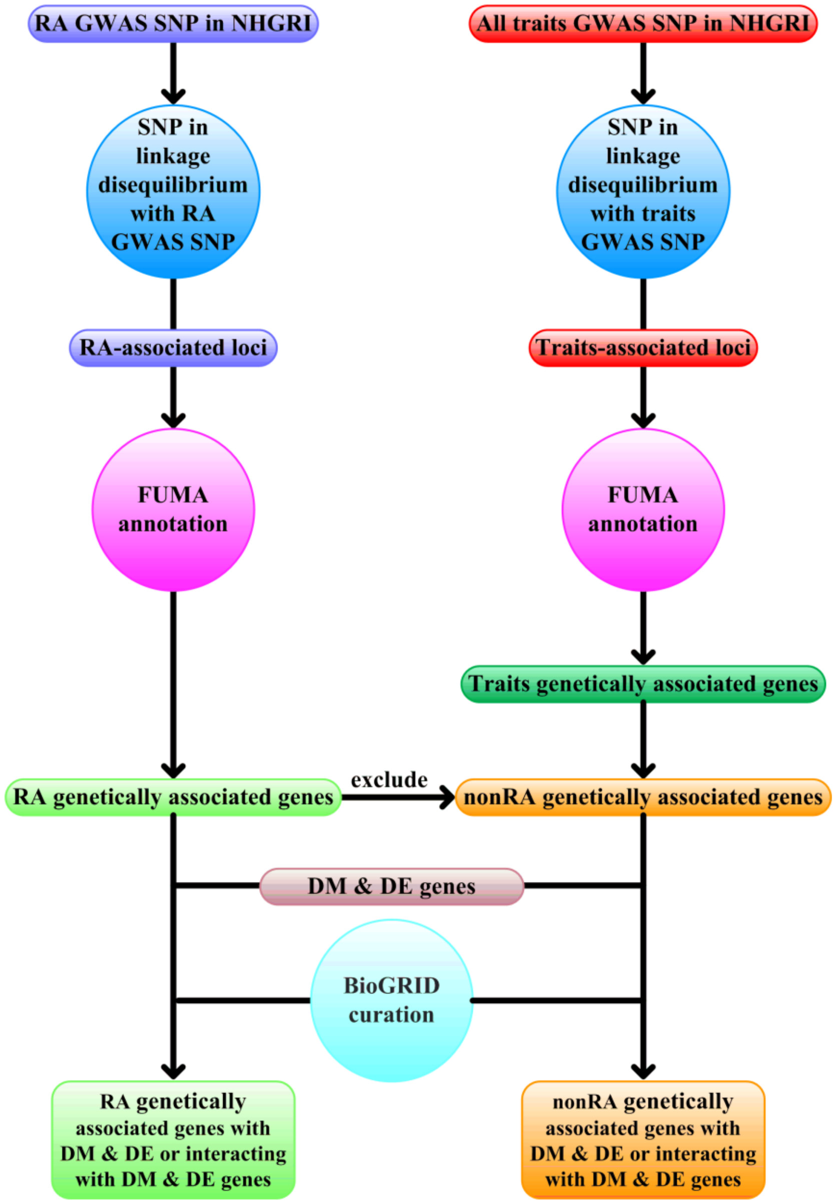

Figure S1. Flowcharts of genetic-epigenetic interaction investigation. RA-associated single nucleotide polymorphisms (SNPs) in past genome-wide association studies (GWAS) (RA GWAS SNP) and SNPs associated with any traits in past GWAS (traits GWAS SNP) were downloaded from NHGRI. SNPs in linkage disequilibrium were identified to yield RA-associated loci and traits-associated loci. After FUMA annotation, RA genetically associated genes and traits genetically associated genes were retrieved. After excluding RA genetically associated genes from traits genetically associated genes, nonRA genetically associated genes were obtained. Intersection of RA genetically associated genes, differentially methylated and differentially expressed genes and BioGRID curation yielded RA genetically associated genes with differential methylation and differential expression or targeting genes with differential methylation and differential expression. Similarly, intersection of nonRA genetically associated genes, differentially methylated and differentially expressed genes and BioGRID curation yielded nonRA genetically associated genes with differential methylation and differential expression or targeting genes with differential methylation and differential expression. DM: differential methylation, DE: differential expression.

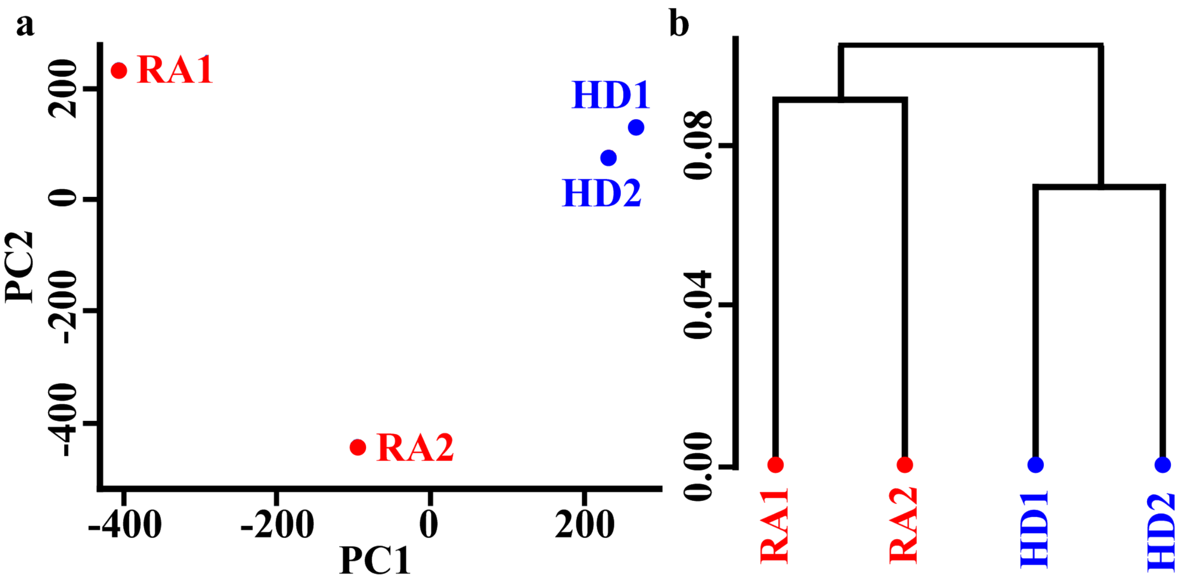

Figure S2. Principal component analysis (PCA) and hierarchical clustering (HC) results. (**a**) PCA plot with samples plotted in two dimensions using their projections onto the first two principal components were shown. Each dot represented a sample. The 2D coordinates of each sample were based on the scores of the first two principal components. (**b**) The dendrogram results of HC (Figure 1, Step 1).

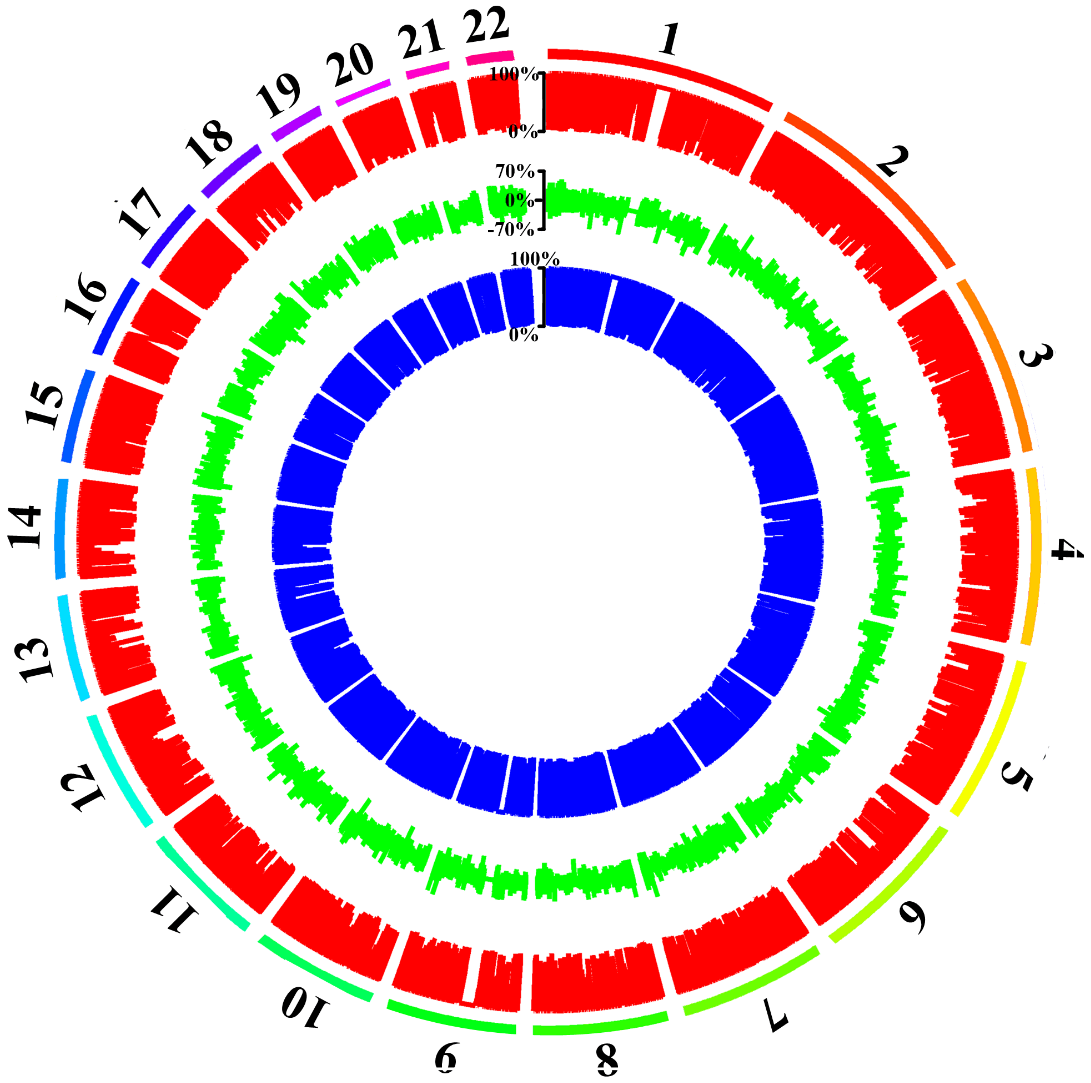

Figure S3. Circular representation of the methylome of rheumatoid arthritis peripheral blood mononuclear cells in different chromosomes**.** Different tracks denoted: methylation level of rheumatoid arthritis peripheral blood mononuclear cells (outer track, red), methylation level of healthy donor peripheral blood mononuclear cells (inner track, blue), and methylation differences (rheumatoid arthritis - healthy donor) between rheumatoid arthritis patients and healthy donors (middle track, green). Positive methylation difference means hypermethylation in rheumatoid arthritis. Conversely, negative methylation difference means hypomethylation in rheumatoid arthritis.

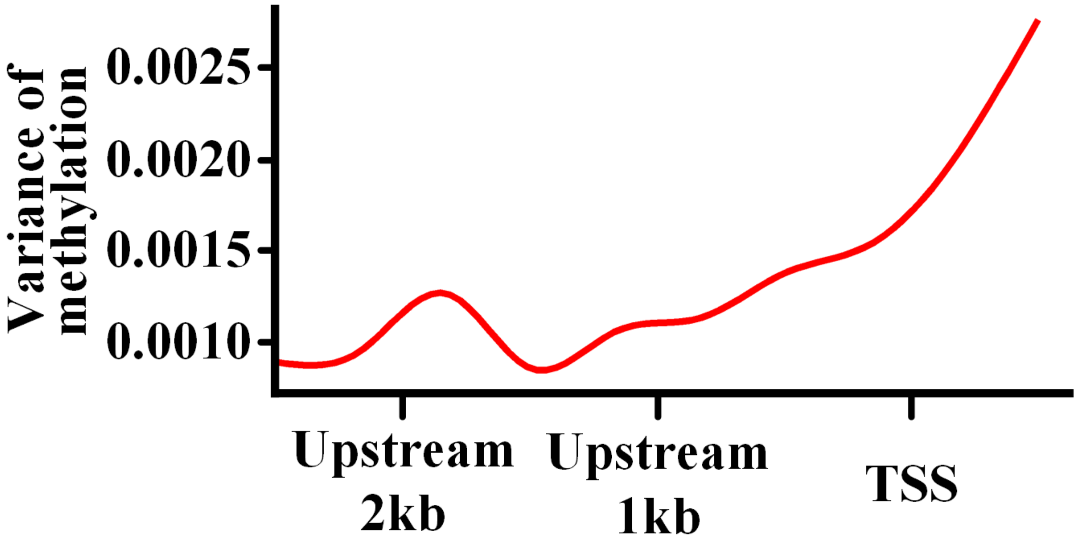

Figure S4. Distribution of methylation variance relative to transcription start sites (TSSs) in promoters. The plot shows the location between 2500 base pair upstream of TSSs and 500 base pair downstream of TSSs. The X-axis represented relative distance (kb) from the TSSs. The Y-axis represented the variance of methylation.

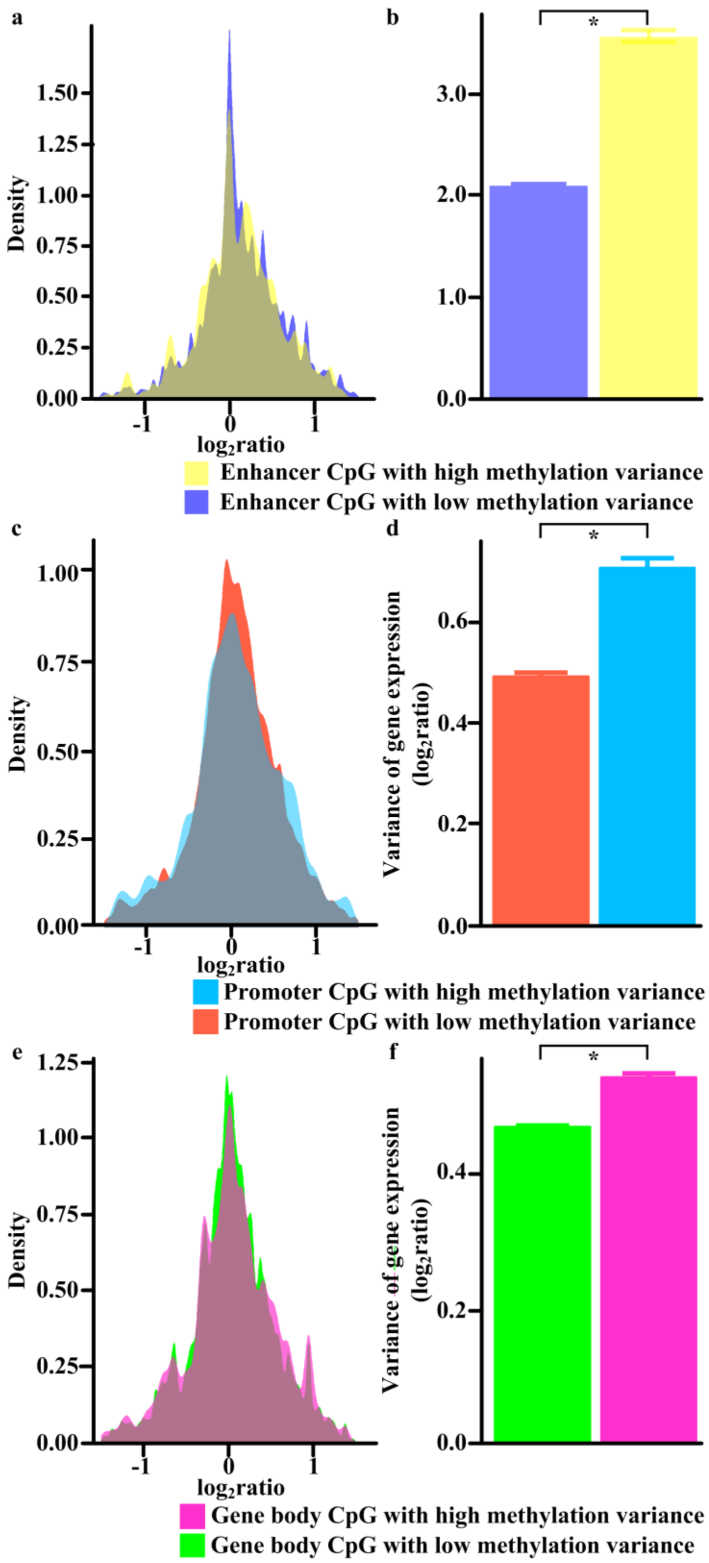

Figure S5. Transcriptional variance was associated with methylation variance at enhancers, promoters and gene bodies. (a) Density plot of log2ratio in transcript levels of enhancer CpG with low (purple) and high (yellow) methylation variance. (b) Barplot of variance in transcript expression from enhancer CpG with low (purple) and high (yellow) methylation variance. (c) Density plot of log2ratio in transcript levels of promoter CpG with low (coral) and high (cyan) methylation variance. (d) Barplot of variance in transcript expression from promoter CpG with low (coral) and high (cyan) methylation variance. (e) Density plot of log2ratio in transcript levels of gene body CpG with low (green) and high (pink) methylation variance. (f) Barplot of variance in transcript expression from gene body CpG with low (green) and high (pink) methylation variance. *: P<0.001.

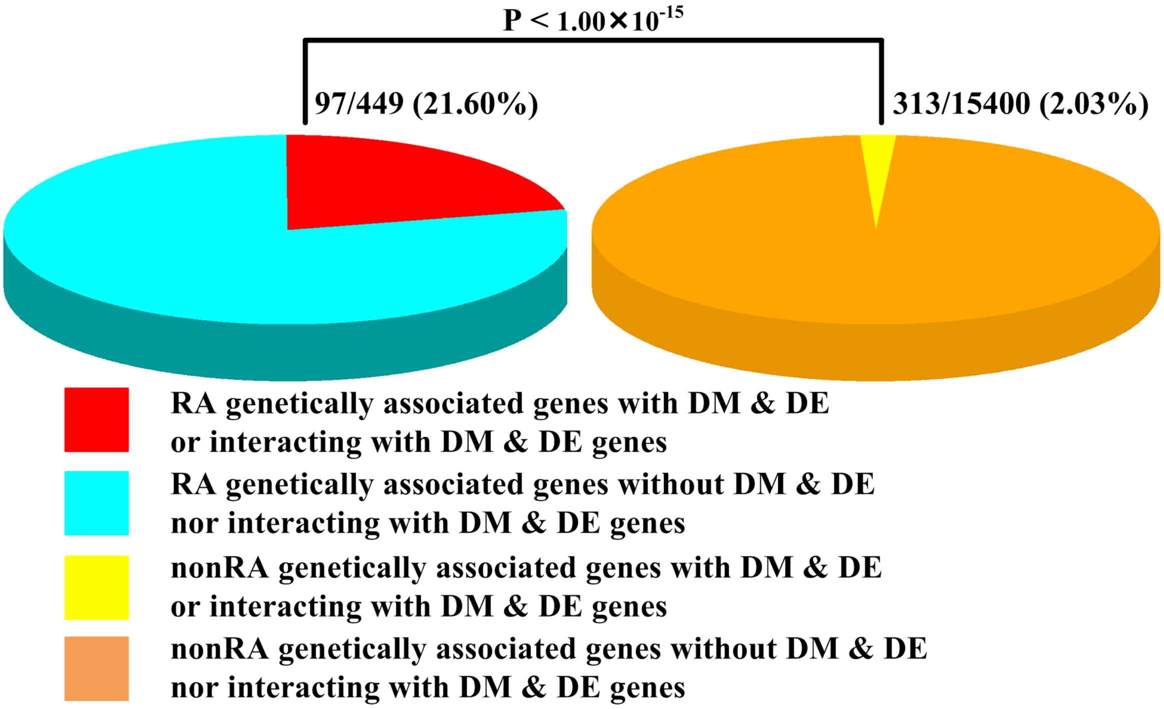

Figure S6. Graphical representation of enrichment of RA genetically associated genes with differential methylation and differential expression or interacting with differentially methylated and differentially expressed genes. Of 449 RA genetically associated genes, 97 (21.60%) displayed differential methylation and differential expression or interacted with differentially methylated and differentially expressed genes, compared with 313/15400 (2.03%) of nonRA genetically associated genes ($P<1.00\times10^{-15}$). DM: differential methylation, DE: differential expression.

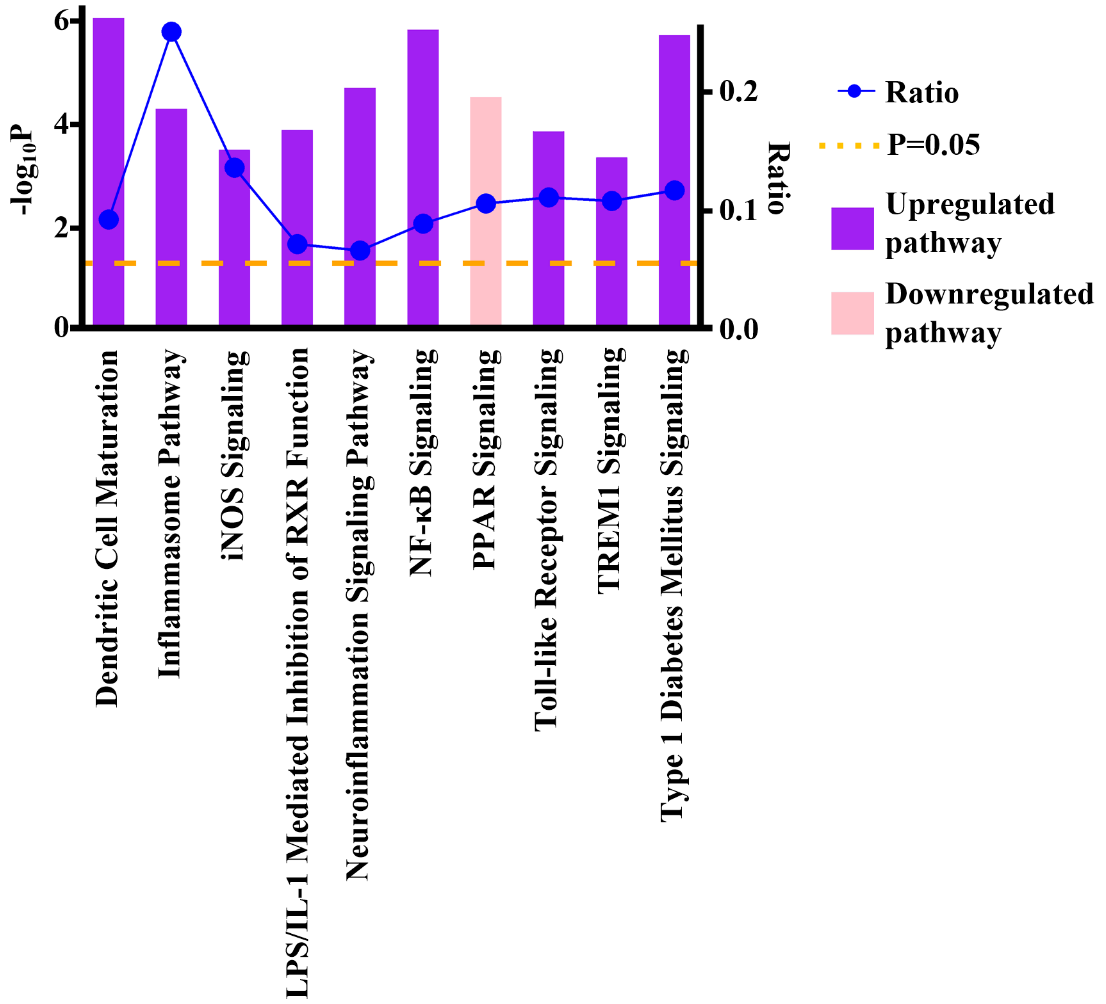

Figure S7. Top 10 significantly perturbed canonical pathways revealed by Ingenuity Pathway Analysis (IPA). The upregulated and downregulated pathways obtained from IPA were shown along the X-axis of the bar chart. The Y-axis indicated the statistical significance on the left. The dashed threshold line represented the default significance cutoff at P=0.05. The blue curve showed the ratio between the number of genes with differential methylation and differential expression and the total number of genes in each of these pathways.

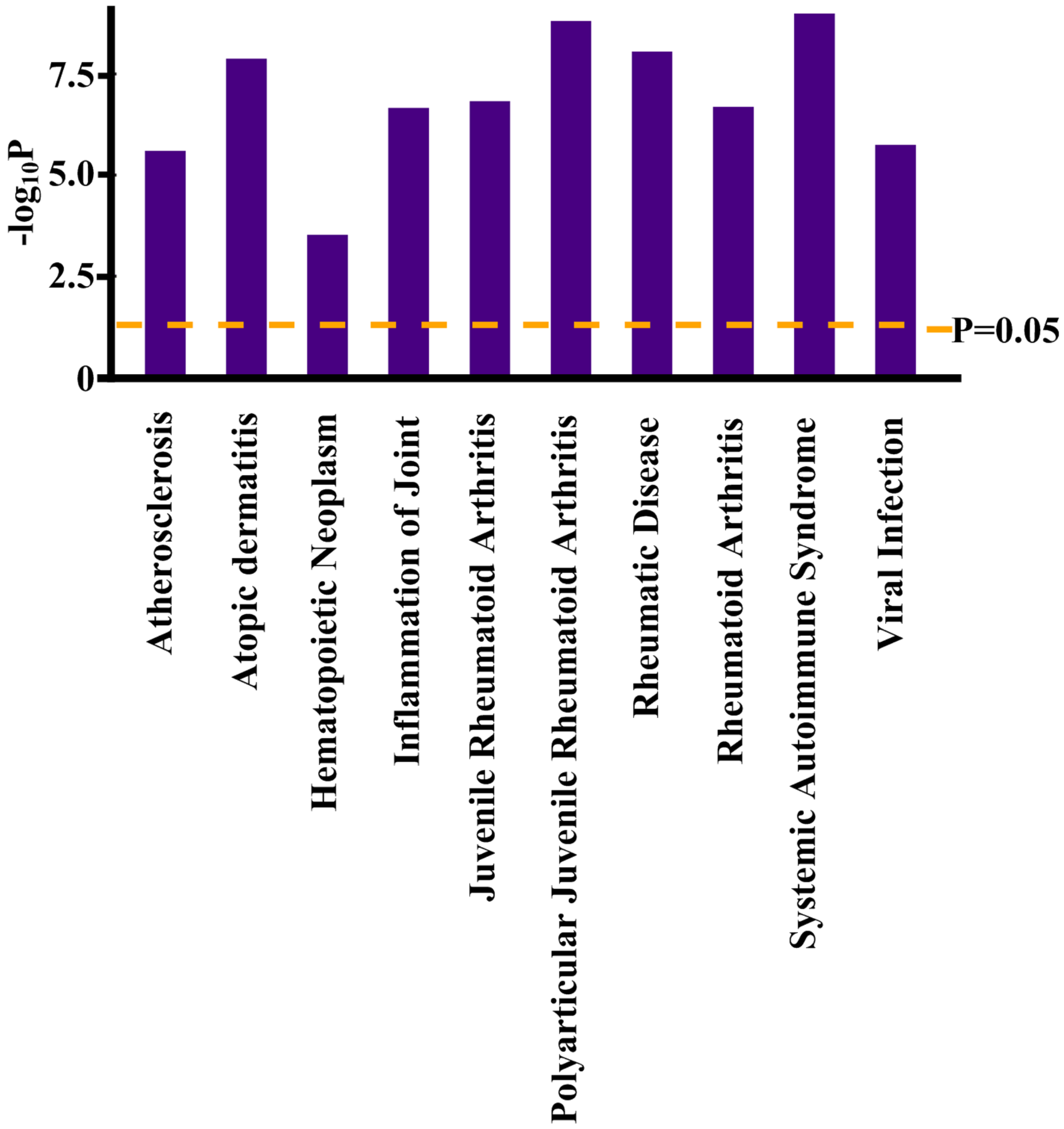

Figure S8. Top 10 diseases associated with genes having concomitant differential methylation and differential expression in rheumatoid arthritis. The dashed line represented a P value of 0.05.

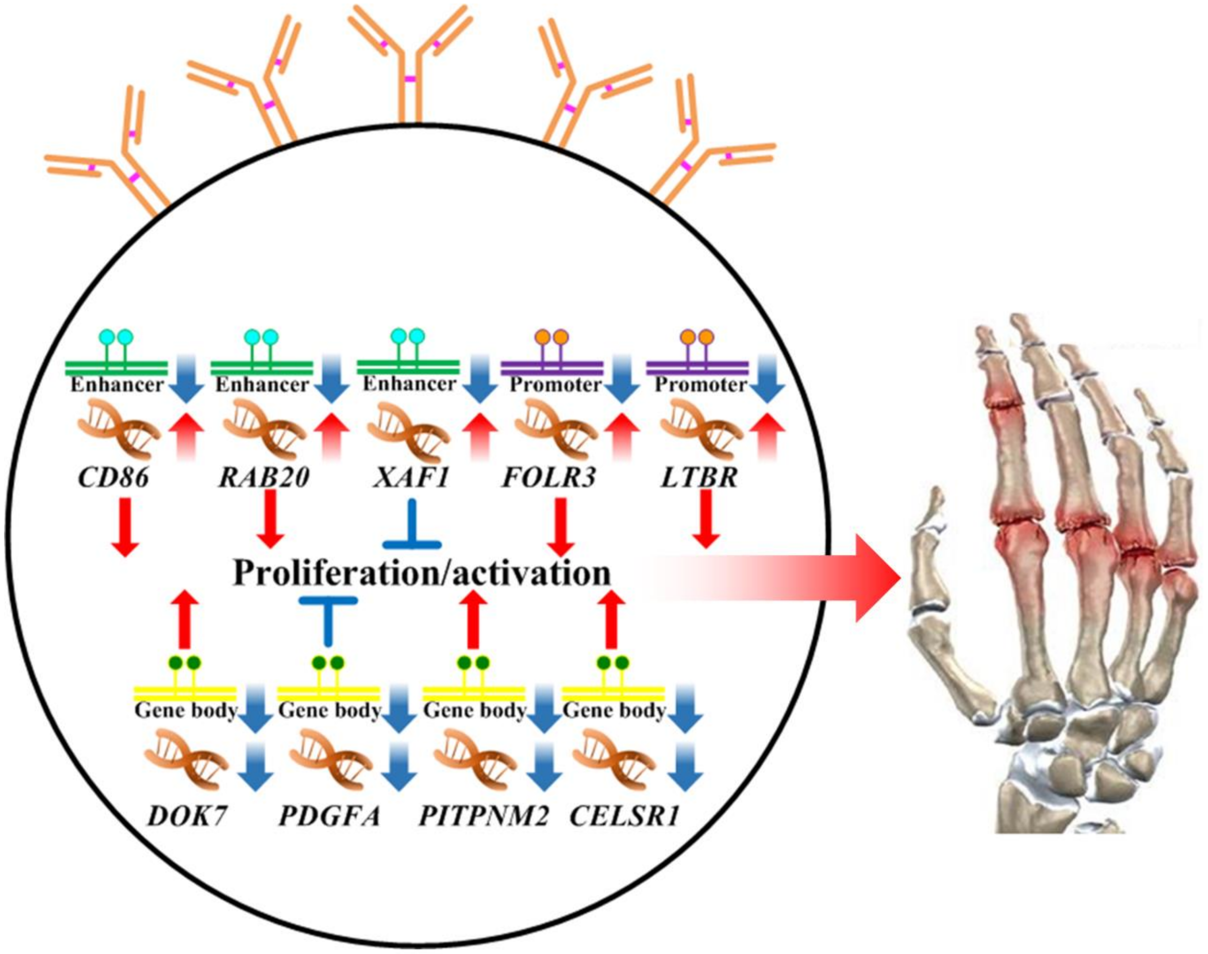

Figure S9. Proposed mechanism of *CD86*, *RAB20*, *XAF1*, *FOLR3*, *LTBR*, *PDGFA*, *DOK7*, *PITPNM2*, *CELSR1* in B cells of rheumatoid arthritis. Enhancer hypomethylation with transcription upregulation of *CD86* and *RAB20* in B cells resulted in immune activation, while enhancer hypomethylation with transcription upregulation of *XAF1* diminished proliferation and thus reduced arthritis. Promoter hypomethylation with transcription upregulation of *FOLR3* and *LTBR* in B cells led to immune activation. Gene body hypomethylation with transcription downregulation of *PDGFA* in B cells inhibited proliferation and thus ameliorated arthritis, while gene body hypomethylation with transcription downregulation of *DOK7*, *PITPNM2*, and *CELSR1* in B cells enhanced proliferation/activation and aggravated arthritis.

© 2019 by the authors. Submitted for possible open access publication under the terms and conditions of the Creative Commons Attribution (CC BY) license (http://creativecommons.org/licenses/by/4.0/).
